# Diagnostic test accuracy of machine learning algorithms for the detection intracranial hemorrhage: a systematic review and meta-analysis study

**DOI:** 10.1186/s12938-023-01172-1

**Published:** 2023-12-04

**Authors:** Masoud Maghami, Shahab Aldin Sattari, Marziyeh Tahmasbi, Pegah Panahi, Javad Mozafari, Kiarash Shirbandi

**Affiliations:** 1 Medical Doctor (MD), School of Medicine, Ahvaz Jundishapur University of Medical Sciences, Ahvaz, Iran; 2 Department of Neurosurgery, Johns Hopkins University School of Medicine, Baltimore, MD USA; 3 Department of Medical Imaging and Radiation Sciences, School of Allied Medical Sciences, Ahvaz Jundishapur University of Medical Sciences, Ahvaz, Iran; 4 Department of Emergency Medicine, School of Medicine, Ahvaz Jundishapur University of Medical Sciences, Ahvaz, Iran; 5 Independent Medical Imaging Researcher, Tehran, Iran; 6 Department of Radiology, Resident (MD), EUREGIO-KLINIK Albert-Schweitzer-Straße GmbH, Nordhorn, Germany

**Keywords:** Brain diseases, Cerebrovascular disorders, Intracranial hemorrhages, Artificial intelligence, Machine learning, Deep learning, Meta-analysis

## Abstract

**Background:**

This systematic review and meta-analysis were conducted to objectively evaluate the evidence of machine learning (ML) in the patient diagnosis of Intracranial Hemorrhage (ICH) on computed tomography (CT) scans.

**Methods:**

Until May 2023, systematic searches were conducted in ISI Web of Science, PubMed, Scopus, Cochrane Library, IEEE Xplore Digital Library, CINAHL, Science Direct, PROSPERO, and EMBASE for studies that evaluated the diagnostic precision of ML model-assisted ICH detection. Patients with and without ICH as the target condition who were receiving CT-Scan were eligible for the research, which used ML algorithms based on radiologists' reports as the gold reference standard. For meta-analysis, pooled sensitivities, specificities, and a summary receiver operating characteristics curve (SROC) were used.

**Results:**

At last, after screening the title, abstract, and full paper, twenty-six retrospective and three prospective, and two retrospective/prospective studies were included. The overall (Diagnostic Test Accuracy) DTA of retrospective studies with a pooled sensitivity was 0.917 (95% CI 0.88–0.943, *I*^2^ = 99%). The pooled specificity was 0.945 (95% CI 0.918–0.964, *I*^2^ = 100%). The pooled diagnostic odds ratio (DOR) was 219.47 (95% CI 104.78–459.66, *I*^2^ = 100%). These results were significant for the specificity of the different network architecture models (*p*-value = 0.0289). However, the results for sensitivity (*p*-value = 0.6417) and DOR (*p*-value = 0.2187) were not significant. The ResNet algorithm has higher pooled specificity than other algorithms with 0.935 (95% CI 0.854–0.973, *I*^2^ = 93%).

**Conclusion:**

This meta-analysis on DTA of ML algorithms for detecting ICH by assessing non-contrast CT-Scans shows the ML has an acceptable performance in diagnosing ICH. Using ResNet in ICH detection remains promising prediction was improved via training in an Architecture Learning Network (ALN).

**Supplementary Information:**

The online version contains supplementary material available at 10.1186/s12938-023-01172-1.

## Background

A potentially fatal disorder known as intracranial hemorrhage (ICH) occurs in 25 per 100,000 yearly, which is related to 2 million strokes globally and has an estimated incidence [1]. There is a variety of fundamental (80–85%) and secondary (15–20%) underlying causes of ICH [2]. The most frequent non-traumatic secondary causes include brain tumors, ischemic strokes, and vascular malformations. Hospital admissions for ICH have grown during the past ten years, primarily because of the elderly population, insufficient blood pressure (BP), and increased use of blood thinners management [3, 4]. In such a way that, rational decrease of BP is an important factor to manage these patients, specifictly for lower than 15 mL ICH volume [5, 6]. The revascularization in the acute phase of strokes can improve the symptoms and better prognosis of these patients [7]. The tissue plasminogen activator (tPA) is the main treatment for ischemic stroke. Moreover, the clot in the blood vessel can be removed by thrombectomy technique that catheter intervent upper of femur; then, using angioplasty blocked artery can be opened up [8, 9].

Neuroimaging is, therefore, essential for the diagnosis of acute ICH because perchance challenging to differentiate it from other diseases, such as ischemic stroke [10]. The successful procedure of a non-contrast computed tomography (CT) for the cerebrum, an accessible and quick technique for diagnosing ICH, are crucial component of the ICH diagnostic process. Fundamental ICH features such as location, edema, ventricular system expansion, and midline shift are morphologically revealed by a CT-Scan [11]. However, more significant CT-Scan usage could delay the identification of ICH, and a growing burden in radiology departments could lead to job-related stress and burnout. In contrast, it has been discovered that artificial intelligence (AI) can improve radiology practice by lowering the amount of effort required [12–14].

Today, the efficiency of machine learning (ML) algorithms, especially improving deep learning (DL) algorithms for computer vision, has advanced significantly. The CT-Scan, one of the most well-known imaging modalities, and has seen considerable breakthroughs in ML and its application [15, 16]. Support vector machine (SVM), Convolutional neural network (CNN), random forest (RF), and conditional random field (CRF) are the most prominent ML algorithms for recognizing brain bleeding from visual data. Even though a great deal of work has already been accomplished in this field, there is still room for growth. Additional research is required to improve the accuracy, precision, and resilience of ML-based brain segmentation [17, 18]. A meta-analysis reported DTA of AI for the detection of ICH; however, this study did not report subgroups for distinguishing between Algorithms and also types of ICH [19]. Therefore, this systematic review and meta-analysis were conducted to objectively evaluate the evidence of ML in the patient diagnosis of ICH on CT scans.

## Results

### Study selection & characteristics

**Fig. 1:**
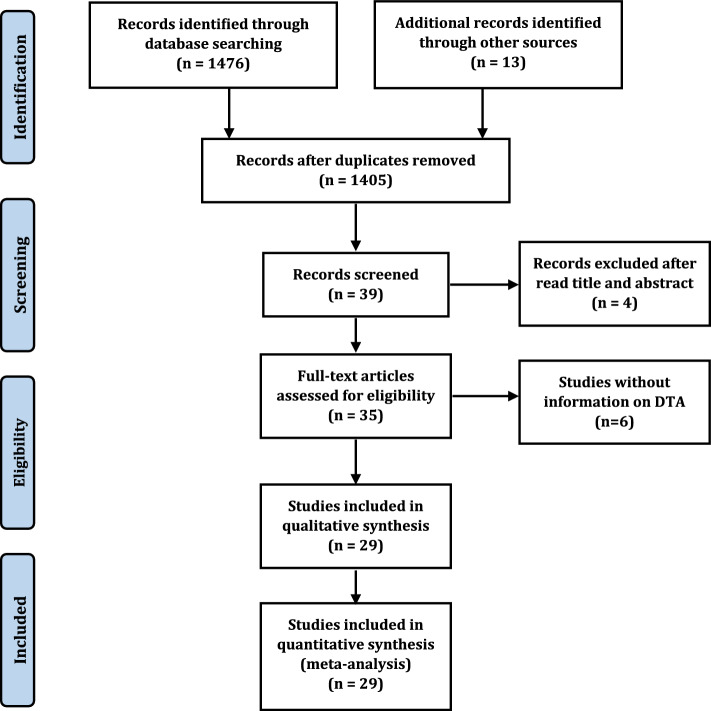
Study Flow Diagram showing how to extract articles

Following the primary search, 1,405 studies were recognized after removing duplicated studies. At last, after screening the title, abstract, and full paper, twenty-six retrospective and three prospective, and two retrospective/prospective studies were included [20–50]; then, twenty-nine studies were included in the final quantitative analysis, and the other studies were excluded because no diagnostic accuracy was reported **(**Fig. 1**)** [20–26, 28–46, 48–52]. The machine learning networks were classified into, Support vector machine (SVM), Random Forest (RF), k-nearest neighbors’ algorithm (k-NN), VGG-16, Logistic Regression (LR), ResNet-18, AlexNet, DenseNet-121, eXtreme Gradient Boosting (XGBoost), Decision Tree (DT), and Deep Learning (DL) included Convolutional Neural Network (CNN); ResNet34, ResNet50, ResNet18, ResNet-v2, GoogleNet **(**Table 1**)**.

**Table 1:** Summary of findings for all studies included in the qualitative synthesis

ID	Study design	ICH type		ML model type	CT-Scan equipment	Data sources	Segmentation	Sensitivity %	Specificity %	Accuracy %	AUC
Schmitt, N., et al. 2022/Germany [39]	Retrospective	ICH		CNN	64-slice multidetector, single-source scanner (Somatom Defnition AS, Siemens Healthineers)	Single/Real-time data	2D	91	89	NA	0.9
Phaphuangwittayakul, A., et al. 2022/China [36]	Retrospective	ICH		CNN	NA	Single/Benchmark	2D	95.77	96.90	96.21	NA
		EDH						95.48	96.02	95.68	
		SDH						96.01	97.55	96.54	
		IPH						95.83	97.13	96.41	
Hopkins, B. S et al. 2022/ USA [29]	Prospective	ICH		DNN	NA	Single/Real-time data	2D	98	99	NA	0.99
Seyam, M., et al. 2022/ Switzerland [40]	Prospective	ICH		DL	256-section scanners (Somatom Force and Somatom Definition Flash, Siemens)	Single/Real-time data	2D	87.2	93.9	93	NA
Altuve, M., & Pérez, A. 2022/Venezuela [22]	Retrospective	ICH		ResNet-18	NA	Single/Real-time data	2D	95.65	96.2	95.93	NA
Tang, Z., et al. 2022/China[41]	Retrospective	ICH		CNN	NA	Single/Real-time data	2D	91.97	88.37	90.58	NA
Cortes-Ferre L, et al. 2022/ Spain [26]	Retrospective	ICH		DL	NA	Single/Benchmark	2D	91.4	94	92.7	0.978
Kau, T., et al. 2022/ Austria [30]	Retrospective	ICH		DL	NA	Single/Real-time data	2D	68.2	96.8	94	NA
Tharek A., et al. 2022/Malaysia [42]	Retrospective	ICH		CNN	NA	Single/Benchmark	2D	96.94	93.14	95	NA
Abe, D., et al. 2022/Japan [20]	Retrospective	ICH		XGBoost	NA	Single/Real-time data	2D	74	74.9	NA	0.8
Trevisi, G.et al. 2022/Italy [43]	Retrospective	ICH		RF	NA	Multiple/Real-time data	2D	77.52	86.29	83.55	0.93
Uchida, K., et al. 2022/Japan [44]	Prospective	ICH	LR	LR	NA	Multiple/Real-time data	2D	43	92	NA	0.82
			RF					41	94		0.82
			XGBoost	RF				40	92		0.81
		SAH	LR					27	97		0.87
			RF	XGBoost				16	98		0.85
			XGBoost					23	97		0.86
Alis, D.. et al. 2022/Turkey [21]	Retrospective	ICH-Binary		CNN-RNN	NA	Multiple/Real-time data	2D	96.41	95.79	96.02	0.961
		IPH						82.56	97.54	94.69	0.905
		IVH						86.84	98.31	97.35	0.925
		SAH						91.67	86.14	86.73	0.889
		SDH						88.16	90.16	89.82	0.891
		EDH						71.4	99.98	98.89	0.98
Rao, B. N. et al. 2022/ India [37]	Retrospective	ICH		VGG-16	64-slice CT scan machine (GE OPTIMA, 64 slice)	Single/Real-time data	2D	91.2	93.1	93.1	0.965
				GoogleNet (InceptionV3)				97.4	98.6	98.9	0.988
				ResNet-50				97.1	99.3	98.2	0.984
				Proposed model							
								99.4	99.7	99.6	1
Zhou, Q., et al. 2022/ China [50]	Retrospective	EDH	ResNet-18	ResNet-18/DenseNet-121	SIEMENS/GE/TOSHIBA/Neusoft	Single/Real-time data	2D	98	88	NA	NA
			DenseNet-121					86	81		
		IVH	ResNet-18					85	91		
			DenseNet-121					73	85		
		CPH	ResNet-18					80	91		
			DenseNet-121					76	84		
		SAH	ResNet-18					81	91		
			DenseNet-121					81	83		
		SDH	ResNet-18					93	89		
			DenseNet-121					85	82		
Salehinejad., H. et al. 2021/Canada [38]	Retrospective	EDH		SE-ResNeXt50-32 and SE-ResNeXt101-32 (DL)	64 row multi-detector CT scanner(Revolution, LightSpeed 64, or Optima 64, General Electric Medical Systems)	Single/Benchmark	2D	21.5	99.9	99.4	60.8
		SDH						84.3	98.5	96.5	91.4
								76.9	98.7	95.5	87.8
								93.2	98.9	97.9	96.0
		SAH						94.1	98.3	97.4	96.2
		IVH									
		IPH									
McLouth J., et al. 2021/USA [35]	Retrospective	Intraparenchymal, Intraventricular, Epidural/Subdural, and Subarachnoid		DL	GE Medical Systems, Philips, Siemens, Canon (Formerly Toshiba), and NMS	Multiple/Real-time data	2D	91.4	97.5	95.6	NA
Voter, Andrew., F et al. 2021/USA [45]	Retrospective	ICH		DSS (DL)	Helical GE, pitch of 0.531, 120 kV, thin axial reconstruction is 1.25-mm slices at 0.625-mm intervals	Multiple/Real-time data	2D	92.3	97.7	NA	NA
Xu J., et al. 2021/China [47]	Retrospective	ICH, EDH, and SDH		Dense U-Net (DL)	NA	Multiple/Real-time data	2D	NA	NA	NA	NA
Danilov, G., et al. 2021/Russian Federation [27]	Retrospective	EDH		ResNexT (DL)	NA	Single/Real-time data	2D	62.6	NA	82.8	0.762
		SDH						51.8	NA	81.8	0.711
		SAH						49.2	NA	82.9	0.748
								72.3	NA	95.2	0.804
		IVH									
								76.6	NA	0.868	0.803
		IPH									
XU X et al. 2021/China [48]	Retrospective	HICH		SVM	NA	Single/Real-time data	2D	90.9	84.1	85	NA
				KNN				90	82.2	83.6	NA
				LR							
				DT				90.9	84.1	85.5	NA
				RF				80	87.5	85.5	NA
								93.3	92.5	92.7	NA
				XGBoost							
								92.3	88.1	89.1	NA
Wang W et al. 2021/China [46]	Retrospective	ICH		2D-CNN	Siemens/SOMATOM Definition AS CT scanner	Multiple/Benchmark	2D	95	94.4	NA	0.988
		EDH						97.4	94	NA	0.984
		IPH						96.5	95.9	NA	0.992
		IVH						97.5	97.4	NA	0.996
		SAH						94	94.2	NA	0.985
		SDH						94.6	93.2	NA	0.983
Kumaravel, P et al. 2021/India [31]	Retrospective	ICH		AlexNet	NA	Multiple/Benchmark	2D	99.35	99.07	99.21	99.96
				AlexNet-SVM							
								99.67	99.53	99.6	99.99
				AlexNet-PCA-SVM				99.58	99.35	99.47	99.98
Ye, H., et al. 2019/ China [49]	Retrospective	ICH		CNN-RNN	NA	Multiple/Real-time data	2D	99	99	99	1
		CPH						92	83	90	0.94
		SAH						69	94	83	0.89
		IVH						84	95	91	0.93
		SDH						86	96	94	0.96
		EDH						69	98	96	0.94
Kuo, W., et al. 2019/USA [32]	Retrospective	ICH		CNN	GE, Siemens	Single/Benchmark	2D	100	90	NA	NA
Lee, H., et al. 2019/USA [33]	Retrospective/Prospective	ICH, IPH, IVH, SDH, EDH or SAH		DCNNs—VGG16, ResNet-50, Inception-v3 and Inception-ResNet-v2 (DL)	NA	Single/Real-time data	2D	ICHr: 98	ICHr: 95	NA	ICHr: 0.993
								IPHr: 92.5	IPHr: 91.8		IPHr: 0.98
								IVHr: 87	IVHr: 95.9		IVHr: 0.979
								SDHr: 87.5	SDHr: 86.9		SDHr: 0.959
								EDHr: 58.3	EDHr: 95.2		EDHr: 0.922
								SAHr: 84.1	SAHr: 88.5		SAHr: 0.96
								ICHp: 92.4	ICHp: 94.9	NA	ICHp: 0.961
								IPHp: 68.8	IPHp: 95		IPHp: 0.921
								IVHp: 83.3	IVHp: 99.5		IVHp: 0.973
								SDHp: 70.5	SDHp: 92.8		SDHp: 0.881
								EDHp: NA	EDHp: NA		EDHp: NA
								SAHp: 76.3	SAHp: 89.9		SAHp: 0.926
Arbabshirani, M. R., et al. 2018/USA [23]	Retrospective	ICH		R-CNN (DL)	17 scanners from 4 different manufacturers	Multiple/Real-time data	2D	70	87	84	0.846
Majumdar, A., et al. 2018/USA [34]	Retrospective	Epidural, Subdural, Subarachnoid, Intraparenchymal		CNN (U-Net)	NA	Single/Real-time data	2D	81	98	NA	NA
Chang, P. D., et al. 2018/USA [24]	Retrospective	ICH		Mask R-CNN + Hybrid 3D/2D CNN	NA	Single/Real-time data	2D	97.1	97.5	NA	0.984
	Prospective							97.5	97.3	NA	0.972
Grewal., et al. 2018/USA[28]	Retrospective	ICH		CNN	NA	Multiple/Benchmark	2D	88.6	72.7	81.82	0.818
Chilamkurthy, S. et al. 2018/India [25]	Retrospective	ICH		CNN transfer learning (ResNet18)		Multiple/Real-time data	2D	98.07	98.73	NA	
		IPH						98.09	98.83		
		IVH						100	100		
		SAH						93.18	99.65		
		EDH						100	99.83		
		SAH									
								100	99.71		

### Risk of bias

**Fig. 2:**
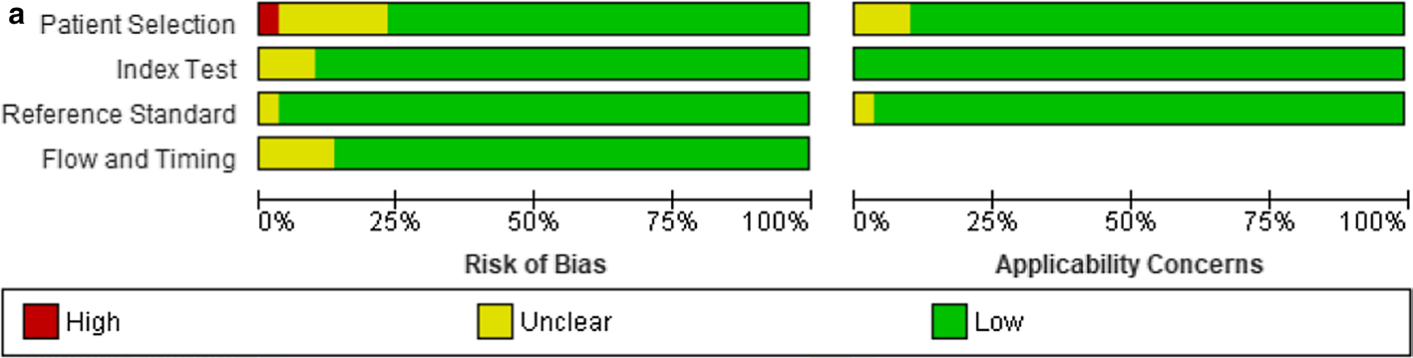

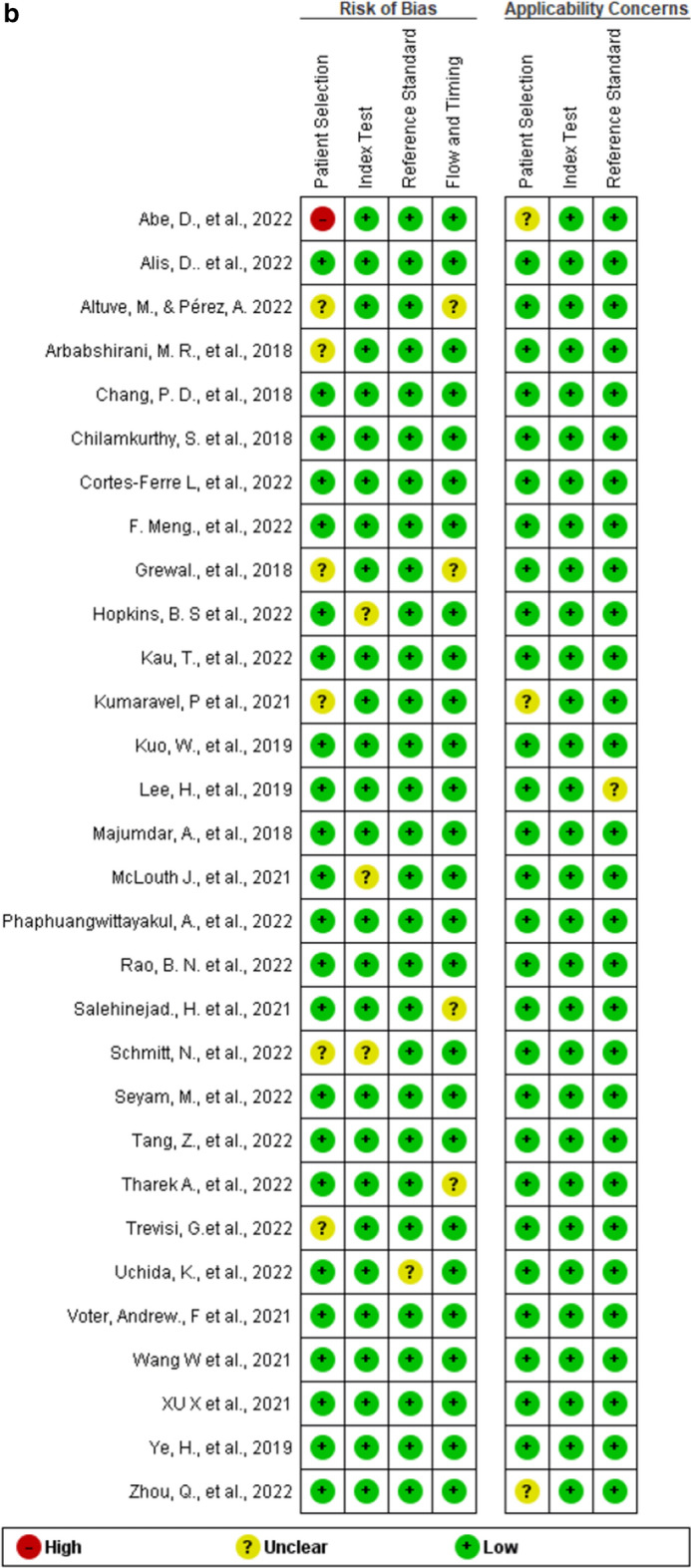
**A**. Risk of bias and applicability concerns graph; review authors' judgments about each domain presented as percentages across included studies. **B.** Risk of bias and applicability concerns summary; review authors' judgments about each domain for each included study

**Fig. 3:**
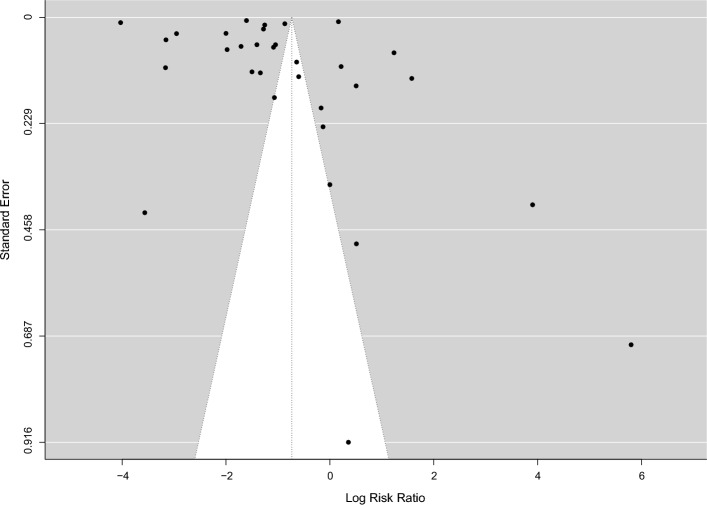
Funnel plot showing the low likelihood of publication bias in all included studies

The validity and the possibility of bias for the included studies were evaluated with the QUADAS-2 (Fig. 2). One high-risk bias was reported in all the included studies [20]. When the publication bias is very low, the points will be symmetrically distributed around the true effect of an inverted funnel, as shown in Fig. 3.

#### Diagnostic test accuracy (DTA) of all included studies

##### Retrospective studies

**Fig. 4:**
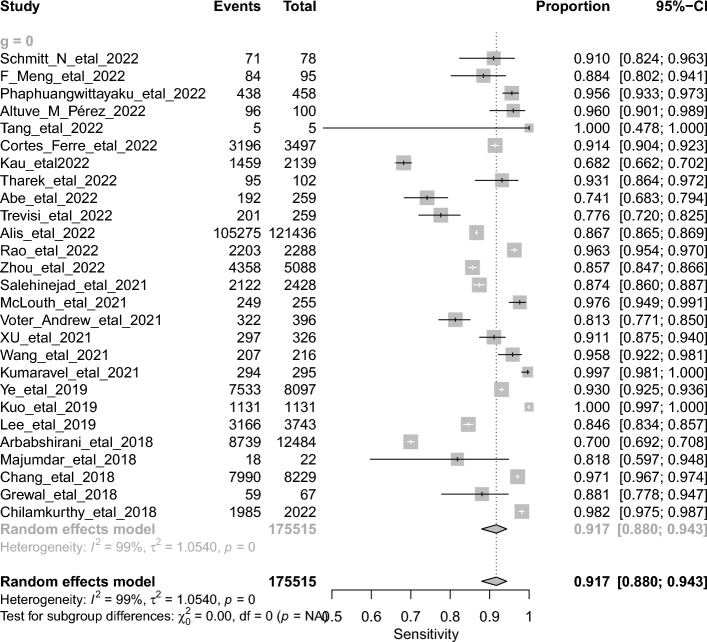
Univariate sub-group analysis of sensitivity with random model based on retrospective studies

**Fig. 5:**
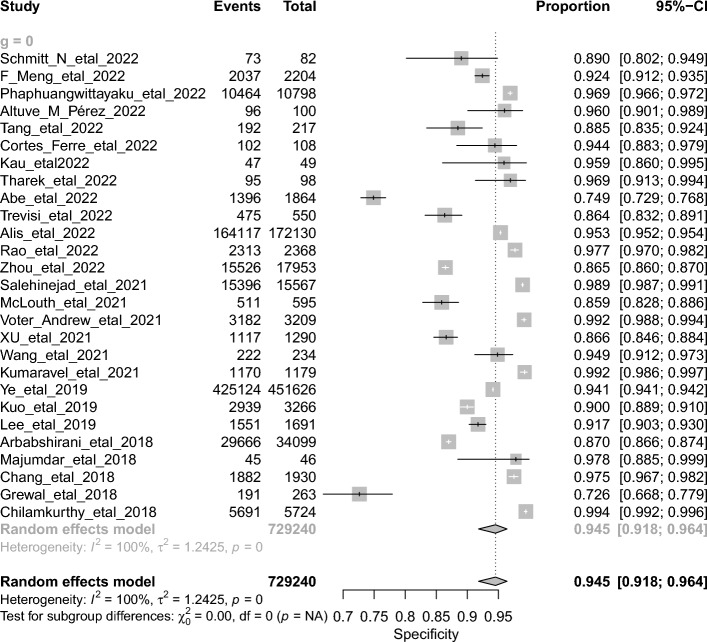
Univariate sub-group analysis of specificity with random model based on retrospective studies

**Fig. 6:**
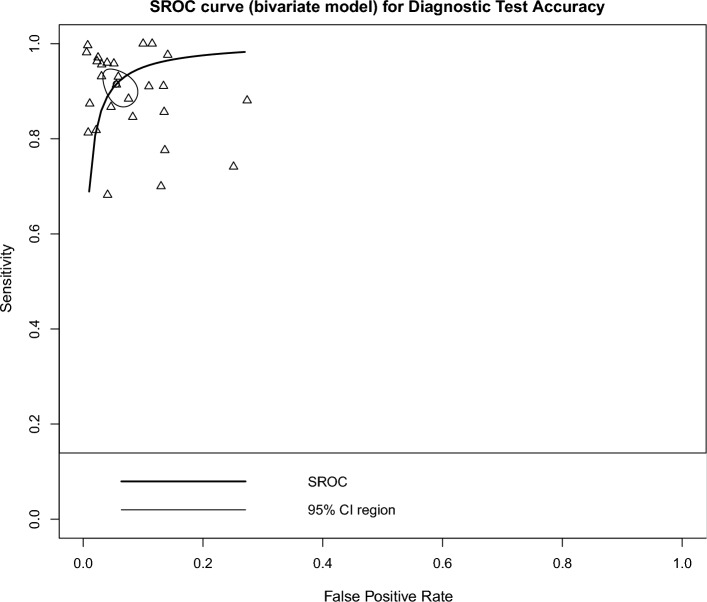
The SROC of the bivariate for DTA based on retrospective studies

The overall DTA of the 26 retrospective studies and 904,755 scans was estimated using a univariate meta-analysis with a pooled sensitivity was 0.917 (95% CI 0.88 to 0.943, *I*^2^ = 99%) **(**Fig. 4**)** [20–26, 28–43, 45, 46, 48–50]. The pooled specificity was 0.945 (95% CI 0.918 to 0.964, *I*^2^ = 100%) **(**Fig. 5**)**. The pooled diagnostic odds ratios (DOR) was 219.47 (95% CI 104.78 to 459.66, *I*^2^ = 100%) **(**Additional file 1: Figure S1). The LR^+^ ranges from 12.639 to 20.784 with pooled mean of 16.208 **(**Table 2**)**, and LR^−^ ranges from 0.072 to 0.123 with pooled mean of 0.094. The AUC of 0.971 was reported for the SROC via the bivariate model **(**Fig. 6**)**. The overall accuracy was 90.3 (ranges from 87.24 to 93.01), the precision was 76.24 (ranges from 66.71 to 86.32), and the F1-score was 79.14 (ranges from 70.9 to 86.48) **(**Table 2**)**.

**Table 2:** DTA estimated from all the studies included in the meta-analysis using (2 $\times$ 2) confusion table

Amount	LR^+^	LR^−^	Accuracy, %	Precision, %	F1-Score
*Retrospective*					
Minimum	12.639	0.072	87.24	66.71	70.9
Maximum	20.784	0.123	93.01	86.32	86.48
Average	16.208	0.094	90.3	76.24	79.14
*Prospective*					
Minimum	6.054	0.005	90.31	55.23	56.23
Maximum	87.029	1.932	97.2	91.18	91.32
Average	22.953	0.101	93.69	75.58	77.26

*DTA* Diagnostic accuracy test

##### Prospective studies

**Fig. 7:**
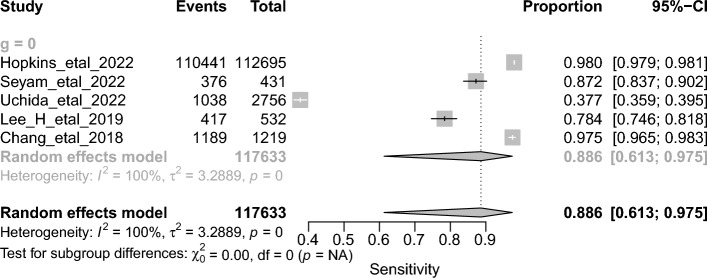
Univariate sub-group analysis of sensitivity with random model based on prospective studies

**Fig. 8:**
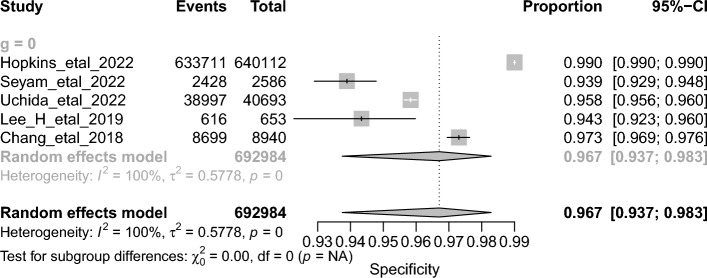
Univariate sub-group analysis of specificity with random model based on prospective studies

**Fig. 9:**
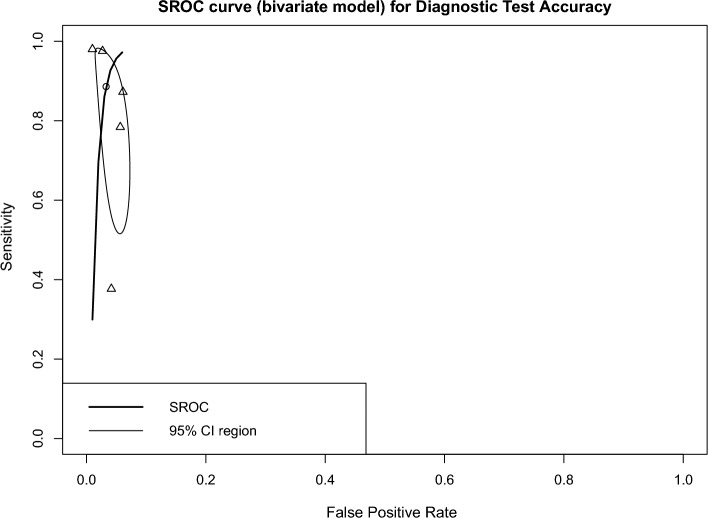
The SROC of the bivariate for DTA based on prospective studies

The overall DTA of the five prospective studies and 104,397 scans was estimated using a univariate meta-analysis with a pooled sensitivity was 0.886 (95% CI 0.613–0.975, *I*^*2*^ = 100%) **(**Fig. 7**)** [24, 29, 33, 40, 44]. The pooled specificity was 0.967 (95% CI 0.937–0.983, *I*^2^ = 100%) **(**Fig. 8**)**. The pooled DOR was 227.71 (95% CI 27.82–1863.51, *I*^2^ = 100%) **(**Additional file 1: Figure S2). The LR^+^ ranges from 6.054 to 87.029 with pooled mean of 22.953 **(**Table 2**)**, and LR^−^ ranges from 0.005 to 1.932 with pooled mean of 0.101. The AUC of 0.98 was reported for the SROC via the bivariate model **(**Fig. 9**)**.

The overall accuracy was 93.69 (ranges from 90.31 to 97.2), the precision was 75.58 (ranges from 55.23 to 91.18), and the F1-score was 77.26 (ranges from 56.23 to 91.32) **(**Table 2**)**.

#### DTA Based on network architecture

The Network Architecture analysis was divided into ResNet, RF, and SVM [20–26, 28, 30–39, 41–43, 45, 46, 48–50]. These results were significant for the specificity of the different network architecture models (*p*-value = 0.0289). However, the results for sensitivity (*p*-value = 0.6417) and DOR (*p*-value = 0.2187) were not significant **(**Additional file 1: Figures S3–S5).

#### DTA based on ICH types

The ICH types of analysis were divided into EDH, SDH, IPH, IVH, SAH, and CPH [21, 25, 33, 36, 38, 46, 49, 50]. These results were significant for the results for specificity (*p*-value < 0.0001) and DOR (*p*-value = 0.0009). However, the sensitivity of different ICH types (*p*-value = 0.4564) was insignificant **(**Additional file 1: Figures S6–S8).

#### DTA based on data sources

The data sources analysis was divided into single [20, 22, 24, 26, 27, 30, 32–34, 36–39, 41, 42, 47, 48, 50] or multiple [21, 23–25, 27, 28, 31, 34, 35, 43, 45–49]. These results were not significant for the sensitivity (p-value = 0.6879), specificity (p-value = 0.6494), and DOR (p-value = 0.7272) **(**Additional file 1: Figures S9–S11).

The data sources analysis was divided into benchmark [26, 28, 31, 32, 36, 38, 42, 46] or real-time data [20–25, 27, 30, 33–35, 37, 39, 41, 43, 45, 47–50]. These results were not significant for the sensitivity (*p*-value = 0.1017), specificity (*p*-value = 0.5189), and DOR (*p*-value = 0.1285) **(**Additional file 1: Figures S12–S14).

## Discussion

Detection of ICH by ML in systematic studies may decrease the time to diagnosis, which is crucial for clinical because approximately most of ICH in accordance with death occurs within the primary hours [53]. This meta-analysis demonstrated that ResNet algorithms could detect ICHs accurately with retrospective and non-randomized data [22, 31, 33, 37, 38, 50].

In this current study, ML has been used in ICH non-contrast CT-Scans with different architecture models. The resulting pooled sensitivity, specificity, DOR, AUC, accuracy, and precision were 0.917 (95% CI 0.88 to 0.943, *I*^2^ = 99%), 0.945 (95% CI 0.918 to 0.964, *I*^2^ = 100%), 219.47 (95% CI 104.78 to 459.66, *I*^2^ = 100%), 0.971, 90.3 (ranges from 87.24 to 93.01), and 76.24 (ranges from 66.71 to 86.32), respectively.

Practical ML is characterized by high accuracy measures such as AUC, sensitivity, and specificity, which can accurately categorize illness suspects and non-suspects. This meta-analysis revealed a combined AUC of 0.971. On the other hand, the high AUC of the included trials could not correctly represent the performance of the algorithm's therapeutic benefit [54]. Initially, the range of AUC among studies was 0.608 to 1 that Neural Networks (NNs) learning such as CNN, ResNet, and RNN had a higher rate from other ML algorithms [20, 21, 23, 24, 26–29, 31, 33, 37–39, 43, 44, 46, 49]. In other words, this result suggested that NNs algorithms in the big data can improve the rate of AUC which it is a useful way to detect a good model and positive and negative target classes.

DL models were shown to have a pooled sensitivity of 87.00% (95% confidence interval: 83.00–90.20%) and specificity of 92.50% (95% confidence interval: 85.10–96.40%) when compared to the gold standard by *Liu *et al*. (2019)*, who pooled 14 out-of-sample external validation experiments [55].

To interpret the results, a DOR of 219.47 (95% CI 104.78–459.66, *I*^2^ = 100%) generally means using ML in diagnosing ICH is valuable. Due to the necessity of reporting the convergence of the results along with the accuracy, precision is also mentioned. Precision equal to 76.24 (ranges from 66.71 to 86.32) indicates a relative convergence besides the accuracy of 90.3 (ranges from 87.24 to 93.01). These results show that ML can be diagnosed with ICH in healthy patients. Also, likelihood ratios are important factors that could help improve clinical judgment and show the range of disease frequencies, and LR^+^ greater than 10 produces a greater pretest probability. The LR^−^ less than 0.1 has conclusive changes in the post-test possibility [56]. The pooled positive LR^+^ and LR^−^ range from 12.639 to 20.784 with a mean of 16.208 and 0.072 to 0.123 with a pooled mean of 0.094, respectively. The pooled LR^+^ of 16.208 means that diagnosis of ICH is 16.208 times more likely to be diagnosed while ML is used; likewise, the pooled LR^−^ of 0.094 means ICH has a higher likelihood of negative test for the ML algorithm than healthy patients. The pooled F1 score of this study was 79.14 (ranging from 70.9 to 86.48). The F1 score is a numerical score between 0 and 100; the closer this number is to 100, the more valuable the method studied [57]. This score results from the average weight of recall and precision, which has a significant place in data interpretation. It can be reduced the number of false negatives and positives.

The sub-group analysis based on the ML architecture and algorithms was done to assess these factors' influence on the DTA results. The network architecture analysis results showed significance for the specificity of the different network architecture models (*p*-value = 0.0289). However, the results for sensitivity (*p*-value = 0.6417) and DOR (*p*-value = 0.2187) were not significant. Thus, the ResNet algorithm has higher pooled specificity than other algorithms 0.935 (95% CI 0.854 to 0.973, *I*^2^ = 93%). Between studies, CNN architectures included specialized neural networks and ensemble learning [58]. However, this study focuses on CNNs for detecting ICHs in general, and it may not be acceptable to extend the results to other AI projects [25]. To increase the number of entirely connected layers from one to five, Lee et al. 2019 combined a final CNN made up of VGG16, ResNet50, Inception-v3, and Inception ResNet-v2 utilizing ResNet18 with only minor alterations [33]. It has been demonstrated that standard ImageNet architectures such as ResNet18 do not significantly outperform smaller and simpler CNNs [59]. However, by averaging many transfer models, the performance of an ensemble of transfer models may be enhanced. Chang et al. (2018) used a hybrid 3D/2D CNN pyramid with a proprietary mask R-CNN architecture as its backbone to detect and segment ICHs [60]. Medical imaging can use finely tuned 3D networks, which have shown exceptional performance in a variety of applications; however, 3D networks need a large dataset and several training parameters, with the image depth volume varying from 20 to 400 slices per scan, which is more demanding in terms of computation efficiency [25].

Besides, the sub-group analysis based on the ICH types was significant for specificity (*p*-value < 0.0001) and DOR (*p*-value = 0.0009). However, the sensitivity of different ICH types (*p*-value = 0.4564) was insignificant. Thus, EDH has higher pooled specificity and DOR than other ICH types 0.99 (95% CI 0.947–0.998, *I*^2^ = 100%) and 616.79 (95% CI 91.76–4145.99, *I*^2^ = 97%). However, there were no significant differences between data sources (single versus multiple or benchmark versus real-time).

Misdetection of ICHs, which are difficult to distinguish from bone or undiscovered microbleeds in trauma imaging, is another therapeutically significant and relevant issue [61]. Using image processing techniques, the skull and face were removed from NCTCs in Kuo et al. 2019 research. They achieved 100% sensitivity in an external test set of 200 NCTCs, which was likely made possible by the simplicity of detecting bleeding when only intracranial structures were considered [62]. Patients excluded or removed because of picture artifacts might improve the algorithm. NCTCs are familiar with patient-related imaging artifacts in CT, such as metallic materials, human movements, and incomplete projections. In addition, the diversity of CT scanners and image reconstruction methods makes direct comparisons between research challenging [33].

## Limitations

Developing a clinical environment where an ML supports the radiologist could improve diagnostic efficacy and should be assessed from a socioeconomic and patient standpoint [63]. The deployment of MLs in clinical operations necessitates a sophisticated configuration coupled with medical imaging systems. Just one of the included articles assessed midline shift [25]. Therefore, this outcome couldn’t analyze. This would be important clinically, as its value > 5 mm may be an indication for urgent neurosurgical review.

Additionally, the findings of the I-squared analysis make it clear that combining the data from these studies may not be appropriate, underscoring the dearth of external validation research. Due to factors like scanning methodology, scanner types, algorithm designs, and reference standards, it is not easy to compare different research, which reduces the generalizability and validity of the findings. The judgment of articles may have been tainted by subjective bias since writers' degrees of experience varied. The creation of additional prospective studies in this area may significantly advance future research since, in addition to the different causes of variability, the use of retrospective studies was the study's most noticeable limitation.

## Conclusion

This meta-analysis on DTA of ML algorithms for detecting ICH by assessing non-contrast CT-Scans shows the ML has an acceptable performance in diagnosing ICH. Using ResNet in ICH detection remains promising prediction was improved via training in an Architecture Learning Network (ALN). However, further studies with greater homogeneity are needed to draw more accurate conclusions about the results of DTA of ML in ICH.

## Methods

### Protocol and registration

This meta-analysis study was reported according to Preferred Reporting Items for Systematic Reviews-Diagnostic Test Accuracy (PRISMA-DTA) guideline [64].

### Eligibility criteria

Original studies were eligible if they met all the following predefined inclusion criteria: a) patients undergoing non-contrast brain computed tomography (CT) scan for the detection of acute or chronic Intracranial hemorrhage (ICH), such as intraparenchymal hemorrhage (IPH), subdural hemorrhage (SDH), epidural hemorrhage (EDH), intraventricular hemorrhage (IVH), and subarachnoid hemorrhage (SAH), or b) using a gold standard (Radiologists) to report the ICH.

### Information sources

Until May 2023, systematic searches were conducted in ISI Web of Science, PubMed, Scopus, Cochrane Library, IEEE Xplore Digital Library, CINAHL, Science Direct, PROSPERO, and EMBASE for studies that evaluated the diagnostic precision of ML model-assisted ICH detection.

### Search strategy

One knowledgeable librarian [KSH] established and refined search tactics through team discussion. “Deep Learning,” “Machine Learning,” “Artificial Intelligence,” “Intracranial Hemorrhages,” “intraparenchymal hemorrhage,” “epidural hemorrhage,” “subdural hemorrhage,” “subarachnoid hemorrhage,” “intraventricular hemorrhage,” “Diagnosis,” “Meta-Analysis,” and “Computerized Tomography” were among the kwywords. Moreover, conferences, editorials, commentaries, reviews, guidelines, book chapters, technical articles, and papers with inadequate citation standards that did not match the conceptual framework of the study were rejected.

### Summary measures

ICHs versus HCs that were true positive (TP, true ICH, predicted to be ICH), true negative (TN, non-ICH predicted to be non-ICH), false positive (FP, non-ICH predicted to be ICH), or false negative (FN, ICH, predicted to be non-ICH) were extracted for meta-analysis purposes. The original study's inclusion criteria were utilized to obtain data for the meta-analysis on detecting ICH. In addition, the publication year, the nation where the research was conducted, the study methodology, the number of patients, and their ages were recovered. The primary outcomes were diagnostic accuracy = ((TP + TN)/(TP + FN + FP + TN)), specificity = TN/(FP + TN), sensitivity = TP/(TP + FN), precision = (TP/TP + FP), F1- Score = 2 × (Precision × Recall/Precision + Recall), negative likelihood ratio (LR^−^) = (1-sensitivity/specificity), positive likelihood ratio (LR^+^) = (sensitivity/1- specificity), DOR = (LR^+^/LR), and the AUC of ML on detecting ICH in the patients, ICH versus healthy controls (HCs) [65, 66]. Comparing the accuracy, sensitivity, and specificity of ML and CT-Scan were the subgroup analysis.

### Risk of bias across studies

Two independent reviewers utilized the updated Quality Assessment of Diagnostic Accuracy Studies (QUADAS-2) instrument to evaluate all studies' quality and potential bias. Communication resolved conflicts, and a third reviewer and reviewers independently assessed the first included papers. Two categories were considered: bias susceptibility and patient selection, index test, and comparative benchmark application. In the flow and pace areas, bias was evaluated.

### Additional analyses

Using the Random Effects Model (RE) technique, a univariate meta-analysis was conducted for each modality's sensitivity and specificity to determine its diagnostic accuracy [67]. The RE model was chosen because of the suspected high proportion of heterogeneity. The primary endpoints were sensitivity, specificity, a summary of receiver operating characteristics (SROC) curve, and diagnostic odds ratio (DOR). Point estimates and 95% confidence intervals (CIs) for each study were calculated to ensure consistency of sensitivity and specificity. A bivariate meta-analysis of sensitivity and specificity used R version 4.1.2 (R Foundation for Statistics Computing, Vienna, Austria, 2021) and RStudio version 1.4.1717 to obtain the SROC curve. This includes the "mada" and "meta" R packages implemented. Then the average AUC of SROC was estimated [68, 69]. The secondary outcomes comprised the positive and negative likelihood ratios, precision, and F1 score. Cochran's Q test and I^2^ statistics were utilized to evaluate statistical heterogeneity between studies. 0–40% indicates insignificant non-uniformity, 30%–60% indicates moderate non-uniformity, and 75–100% indicates considerable non-uniformity for Q statistics. A funnel chart was used to examine and depict publication bias (32). All p-values are derived from two-sided tests, and *p*-values of 0.05 are statistically significant. Screening based on machine learning algorithms, ICH types, retrospective or prospective study design, and acute or chronic ICHs was used to perform subgroup analysis. Using the Cochrane Review Manager version 5.4 (RevMan 5.4) program, bias cross-study risk and applicability concern charts were assessed.

### Supplementary Information

**Additional file 1: Figure S1.** Univariate sub-group analysis of DOR with random model based on retrospective studies. **Figure S2.** Univariate sub-group analysis of DOR with random model based on prospective studies. **Figure S3.** Univariate sub-group analysis of specificity with random model based on Network Architecture. G represents sub-group analysis of data, when g = 0 (CNN), g = 1 (ResNet), g = 2 (RF), and g = 3 (SVM). **Figure S4.** Univariate sub-group analysis of sensitivity with random model based on Network Architecture. G represents sub-group analysis of data, when g = 0 (CNN), g = 1 (ResNet), g = 2 (RF), and g = 3 (SVM). **Figure S5.** Univariate sub-group analysis of DOR with random model based on Network Architecture. G represents sub-group analysis of data, when g = 0 (CNN), g = 1 (ResNet), g = 2 (RF), and g = 3 (SVM). **Figure S6.** Univariate sub-group analysis of specificity with random model based on ICH types. G represents sub-group analysis of data, when g = 0 (EDH), g = 1 (SDH), g = 2 (IPH), g = 3 (IVH), g = 4 (SAH), and g = 5 (CPH). **Figure S7.** Univariate sub-group analysis of DOR with random model based on ICH types. G represents sub-group analysis of data, when g = 0 (EDH), g = 1 (SDH), g = 2 (IPH), g = 3 (IVH), g = 4 (SAH), and g = 5 (CPH). **Figure S8.** Univariate sub-group analysis of sensitivity with random model based on ICH types. G represents sub-group analysis of data, when g = 0 (EDH), g = 1 (SDH), g = 2 (IPH), g = 3 (IVH), g = 4 (SAH), and g = 5 (CPH). **Figure S9.** Univariate sub-group analysis of sensitivity with random model based on single or multiple center. G represents sub-group analysis of data, when g = 0 (Single), and g = 1 (Multiple). **Figure S10.** Univariate sub-group analysis of specificity with random model based on single or multiple center. G represents sub-group analysis of data, when g = 0 (Single), and g = 1 (Multiple). **Figure S11.** Univariate sub-group analysis of DOR with random model based on single or multiple center. G represents sub-group analysis of data, when g = 0 (Single), and g = 1 (Multiple). **Figure S12.** Univariate sub-group analysis of sensitivity with random model based on benchmark or real-time data. G represents sub-group analysis of data, when g = 0 (benchmark), and g = 1 (real-time data). **Figure S13.** Univariate sub-group analysis of specificity with random model based on benchmark or real-time data. G represents sub-group analysis of data, when g = 0 (benchmark), and g = 1 (real-time data). **Figure S14.** Univariate sub-group analysis of DOR with random model based on benchmark or real-time data. G represents sub-group analysis of data, when g = 0 (benchmark), and g = 1 (real-time data).

## Data Availability

Not applicable.

## References

[1] An SJ, Kim TJ, Yoon BW (2017). Epidemiology, risk factors, and clinical features of intracerebral hemorrhage: an update. J Stroke.

[2] Rindler RS (2020). Neuroimaging of intracerebral hemorrhage. Neurosurgery.

[3] Hong JM, Kim DS, Kim M (2021). Hemorrhagic transformation after ischemic stroke: mechanisms and management. Front Neurol.

[4] Ginat DT (2020). Analysis of head CT scans flagged by deep learning software for acute intracranial hemorrhage. Neuroradiology.

[5] Shi L (2017). Blood pressure management for acute intracerebral hemorrhage: a meta-analysis. Sci Rep.

[6] Rabinstein AA (2018). Optimal Blood Pressure After Intracerebral Hemorrhage: Still a Moving Target. Stroke.

[7] Rha JH, Saver JL (2007). The impact of recanalization on ischemic stroke outcome: a meta-analysis. Stroke.

[8] Leng T, Xiong ZG (2019). Treatment for ischemic stroke: From thrombolysis to thrombectomy and remaining challenges. Brain Circ.

[9] Hughes RE, Tadi P, Bollu PC (2023). TPA Therapy. StatPearls.

[10] Sporns PB (2021). Neuroimaging of acute intracerebral hemorrhage. J Clin Med.

[11] Vidhya V (2021). Automated detection and screening of traumatic brain injury (TBI) using computed tomography images: a comprehensive review and future perspectives. Int J Environ Res Public Health.

[12] Rao B (2021). Utility of artificial intelligence tool as a prospective radiology peer reviewer—detection of unreported intracranial hemorrhage. Acad Radiol.

[13] Hosny A (2018). Artificial intelligence in radiology. Nat Rev Cancer.

[14] Derevianko A (2023). The use of artificial intelligence (AI) in the radiology field: what is the state of doctor0patient communication in cancer diagnosis?. Cancers.

[15] Rana M, Bhushan M (2022). Machine learning and deep learning approach for medical image analysis: diagnosis to detection. Multimed Tools Appl.

[16] Sarker IH (2021). Machine learning: algorithms, real-world applications and research directions. SN Comput Sci.

[17] Lee JY (2020). Detection and classification of intracranial haemorrhage on CT images using a novel deep-learning algorithm. Sci Rep.

[18] Kundisch A (2021). Deep learning algorithm in detecting intracranial hemorrhages on emergency computed tomographies. PLoS ONE.

[19] Matsoukas S (2022). Accuracy of artificial intelligence for the detection of intracranial hemorrhage and chronic cerebral microbleeds: a systematic review and pooled analysis. Radiol Med.

[20] Abe D (2022). A prehospital triage system to detect traumatic intracranial hemorrhage using machine learning algorithms. JAMA Netw Open.

[21] Alis D (2022). A joint convolutional-recurrent neural network with an attention mechanism for detecting intracranial hemorrhage on noncontrast head CT. Sci Rep.

[22] Altuve M, Pérez A (2022). Intracerebral hemorrhage detection on computed tomography images using a residual neural network. Phys Med.

[23] Arbabshirani MR (2018). Advanced machine learning in action: identification of intracranial hemorrhage on computed tomography scans of the head with clinical workflow integration. NPJ Digit Med.

[24] Chang PD (2018). Hybrid 3D/2D convolutional neural network for hemorrhage evaluation on head CT. AJNR Am J Neuroradiol.

[25] Chilamkurthy S (2018). Deep learning algorithms for detection of critical findings in head CT scans: a retrospective study. Lancet.

[26] Cortes-Ferre L (2022). Deep Learning Applied to Intracranial Hemorrhage Detection. J Imaging.

[27] Danilov G (2020). Classification of Intracranial Hemorrhage Subtypes Using Deep Learning on CT Scans. Stud Health Technol Inform.

[28] Grewal M (2018). Radnet: Radiologist level accuracy using deep learning for hemorrhage detection in ct scans in 2018 IEEE 15th International symposium on biomedical imaging (ISBI 2018). IEEE.

[29] Hopkins BS (2022). Mass deployment of deep neural network: real-time proof of concept with screening of intracranial hemorrhage using an open data set. Neurosurgery.

[30] Kau T (2022). FDA-approved deep learning software application versus radiologists with different levels of expertise: detection of intracranial hemorrhage in a retrospective single-center study. Neuroradiology.

[31] Kumaravel P (2021). A simplified framework for the detection of intracranial hemorrhage in CT brain images using deep learning. Curr Med Imaging.

[32] Kuo W (2019). Expert-level detection of acute intracranial hemorrhage on head computed tomography using deep learning. Proc Natl Acad Sci U S A.

[33] Lee H (2019). An explainable deep-learning algorithm for the detection of acute intracranial haemorrhage from small datasets. Nat Biomed Eng.

[34] Majumdar A (2018). Detecting Intracranial Hemorrhage with Deep Learning. Annu Int Conf IEEE Eng Med Biol Soc.

[35] McLouth J (2021). Validation of a deep learning tool in the detection of intracranial hemorrhage and large vessel occlusion. Front Neurol.

[36] Phaphuangwittayakul A (2022). An optimal deep learning framework for multi-type hemorrhagic lesions detection and quantification in head CT images for traumatic brain injury. Appl Intell.

[37] Rao BN (2022). Deep transfer learning for automatic prediction of hemorrhagic stroke on CT images. Comput Math Methods Med.

[38] Salehinejad H (2021). A real-world demonstration of machine learning generalizability in the detection of intracranial hemorrhage on head computerized tomography. Sci Rep.

[39] Schmitt N (2022). Automated detection and segmentation of intracranial hemorrhage suspect hyperdensities in non-contrast-enhanced CT scans of acute stroke patients. Eur Radiol.

[40] Seyam M (2022). Utilization of artificial intelligence-based intracranial hemorrhage detection on emergent noncontrast CT images in clinical workflow. Radiol Artif Intell.

[41] Tang Z (2022). Deep learning-based prediction of hematoma expansion using a single brain computed tomographic slice in patients with spontaneous intracerebral hemorrhages. World Neurosurg.

[42] Tharek A (2022). Intracranial hemorrhage detection in CT scan using deep learning. Asian J Med Technol.

[43] Trevisi G (2022). Machine learning model prediction of 6-month functional outcome in elderly patients with intracerebral hemorrhage. Neurosurg Rev.

[44] Uchida K (2022). Development of machine learning models to predict probabilities and types of stroke at prehospital stage: the Japan urgent stroke triage score using machine learning (JUST-ML). Transl Stroke Res.

[45] Voter AF (2021). Diagnostic accuracy and failure mode analysis of a deep learning algorithm for the detection of intracranial hemorrhage. J Am Coll Radiol.

[46] Wang X (2021). A deep learning algorithm for automatic detection and classification of acute intracranial hemorrhages in head CT scans. NeuroImage Clinical.

[47] Xu J (2021). Deep network for the automatic segmentation and quantification of intracranial hemorrhage on CT. Front Neurosci.

[48] Xu X (2021). Prognostic prediction of hypertensive intracerebral hemorrhage using CT radiomics and machine learning. Brain and behavior.

[49] Ye H (2019). Precise diagnosis of intracranial hemorrhage and subtypes using a three-dimensional joint convolutional and recurrent neural network. Eur Radiol.

[50] Zhou Q (2022). Transfer learning of the ResNet-18 and DenseNet-121 model used to diagnose intracranial hemorrhage in CT scanning. Curr Pharm Des.

[51] Neves G (2023). External validation of an artificial intelligence device for intracranial hemorrhage detection. World Neurosurg.

[52] Abrigo JM (2023). Artificial intelligence for detection of intracranial haemorrhage on head computed tomography scans: diagnostic accuracy in Hong Kong. Hong Kong Med J.

[53] O'Neill TJ (2021). Active reprioritization of the reading worklist using artificial intelligence has a beneficial effect on the turnaround time for interpretation of head CT with intracranial hemorrhage. Radiol Artif Intell.

[54] Fleming TR, DeMets DL (1996). Surrogate end points in clinical trials: are we being misled?. Ann Intern Med.

[55] Liu X (2019). A comparison of deep learning performance against health-care professionals in detecting diseases from medical imaging: a systematic review and meta-analysis. Lancet Digit Health.

[56] Jaeschke R, Guyatt GH, Sackett DL (1994). Users' guides to the medical literature III. How to use an article about a diagnostic test B. What are the results and will they help me in caring for my patients? The evidence-based medicine working group. JAMA.

[57] Goutte C, Gaussier E (2005). A probabilistic interpretation of precision, recall and F-score, with implication for evaluation. in European conference on information retrieval.

[58] Daugaard Jorgensen M (2022). Convolutional neural network performance compared to radiologists in detecting intracranial hemorrhage from brain computed tomography: A systematic review and meta-analysis. Eur J Radiol.

[59] Raghu M (2019). Transfusion: Understanding transfer learning for medical imaging. Adv Neural Inf Process Syst.

[60] Singh SP (2020). 3D deep learning on medical images: a review. Sensors.

[61] Samek W (2017). Evaluating the visualization of what a deep neural network has learned. IEEE Trans Neural Netw Learn Syst.

[62] Barrett JF, Keat N (2004). Artifacts in CT: recognition and avoidance. Radiographics.

[63] Nagendran M (2020). Artificial intelligence versus clinicians: systematic review of design, reporting standards, and claims of deep learning studies. BMJ.

[64] McInnes MDF (2018). Preferred reporting items for a systematic review and meta-analysis of diagnostic test accuracy studies: the PRISMA-DTA statement. JAMA.

[65] Cronin P (2018). How to Perform a Systematic Review and Meta-analysis of Diagnostic Imaging Studies. Acad Radiol.

[66] Manikandan R, Dorairajan LN (2011). How to appraise a diagnostic test. Indian J Urol.

[67] Shim SR, Kim SJ, Lee J (2019). Diagnostic test accuracy: application and practice using R software. Epidemiol Health.

[68] Doebler P, Holling H (2015). Meta-analysis of diagnostic accuracy with mada. R Packag.

[69] Guo J, Riebler A (2015). meta4diag: Bayesian bivariate meta-analysis of diagnostic test studies for routine practice. arXiv Prepr.

