# Supplementary material for: Diagnostic test accuracy of machine learning algorithms for the detection intracranial hemorrhage: a systematic review and meta-analysis study
Source: Biomed Eng Online. 2023 Dec 4;22:114. doi: 10.1186/s12938-023-01172-1 (PMC10694901; doi:10.1186/s12938-023-01172-1)

Figure S1. Univariate sub-group analysis of DOR with random model based on retrospective studies.

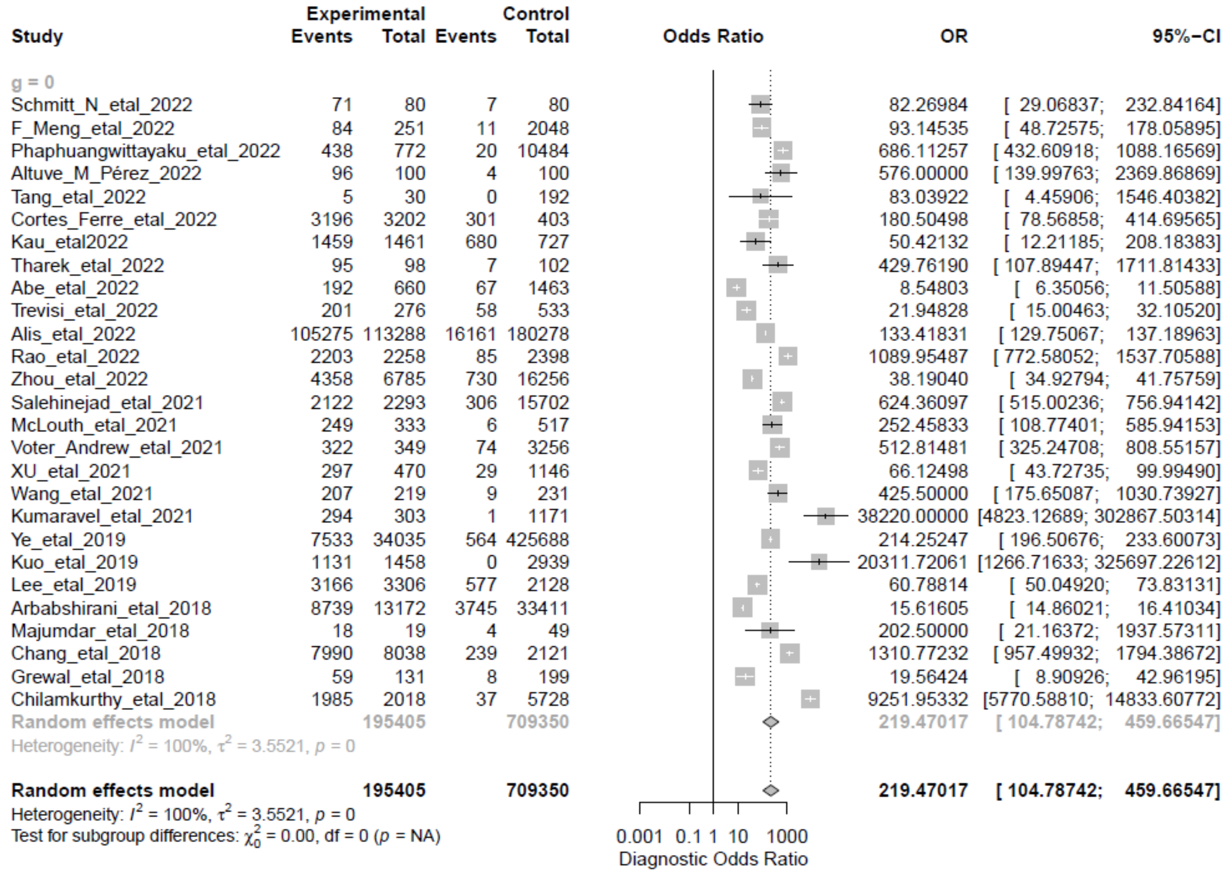

Figure S2. Univariate sub-group analysis of DOR with random model based on proospective studies.

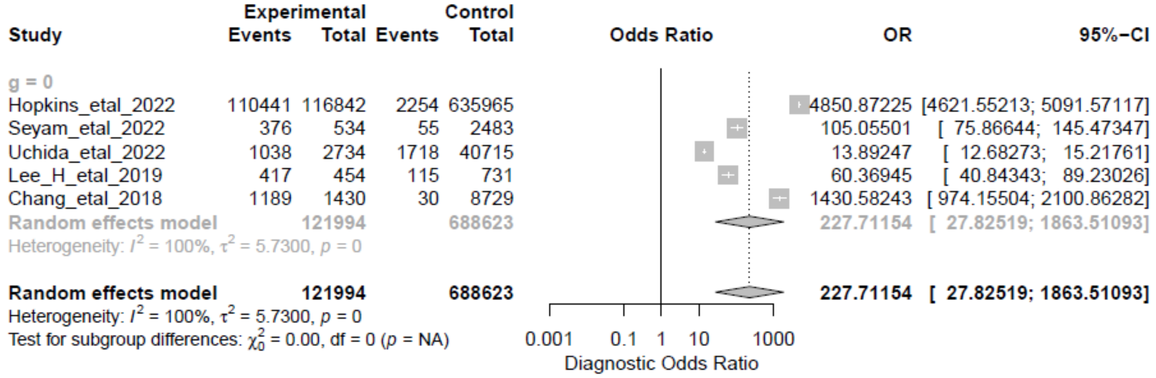

Figure S3. Univariate sub-group analysis of specificity with random model based on Network Architecture. G represents sub-group analysis of data, when g = 0 (CNN), g = 1 (ResNet), g = 2 (RF), and g = 3 (SVM).

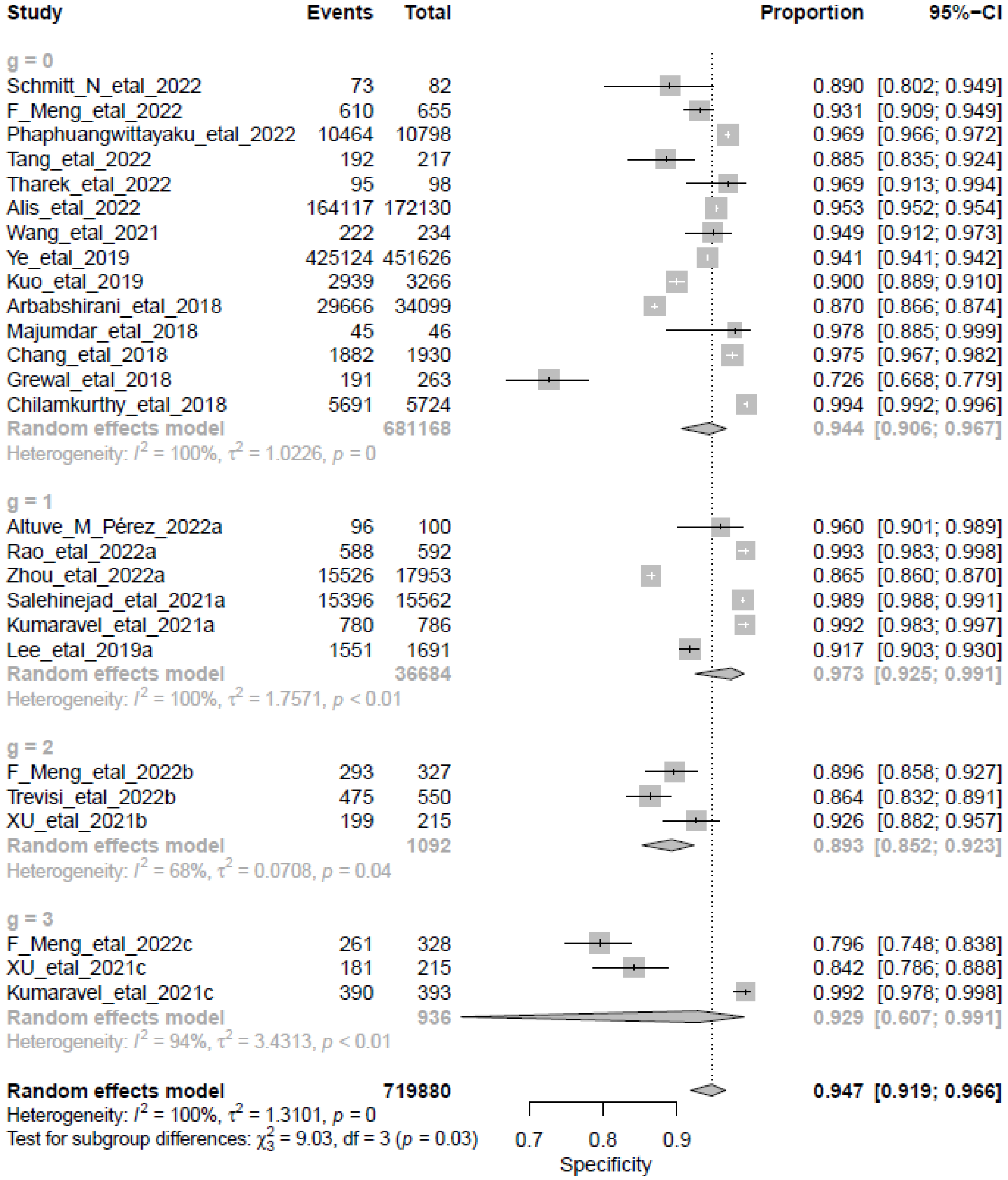

Figure S4. Univariate sub-group analysis of sensitivity with random model based on Network Architecture. G represents sub-group analysis of data, when g = 0 (CNN), g = 1 (ResNet), g = 2 (RF), and g = 3 (SVM).

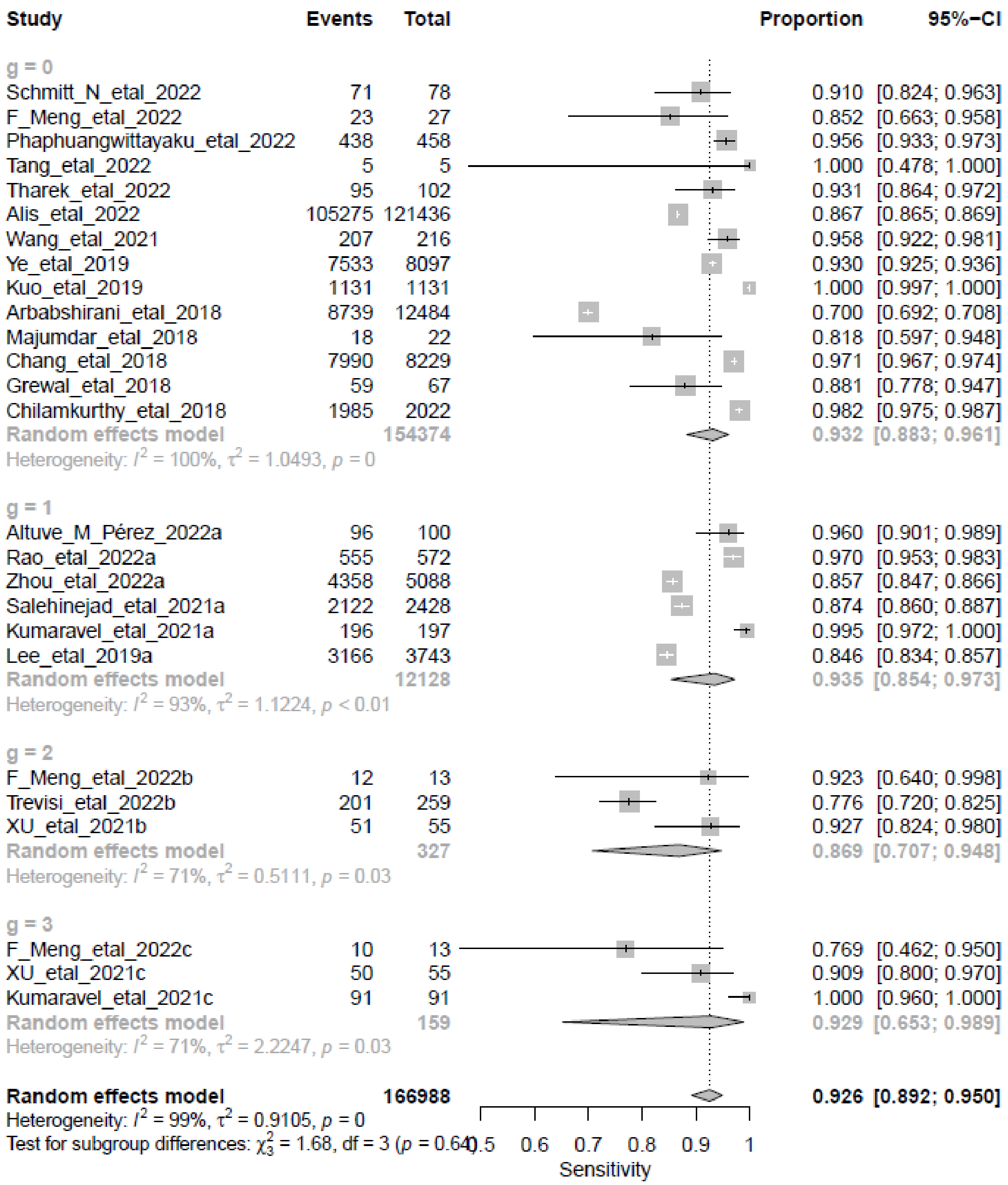

Figure S5. Univariate sub-group analysis of DOR with random model based on Network Architecture. G represents sub-group analysis of data, when g = 0 (CNN), g = 1 (ResNet), g = 2 (RF), and g = 3 (SVM).

Study	Experimental Events	Experimental Total	Control Events	Control Total	OR	95%-CI
g = 0						
Schmitt_N_etal_2022	71	80	7	80	82.26984	[29.06837; 232.84164]
F_Meng_etal_2022	23	68	4	614	77.94444	[25.83916; 235.12125]
Phaphuangwittayaku_etal_2022	438	772	20	10484	686.11257	[432.60918; 1088.16569]
Tang_etal_2022	5	30	0	192	83.03922	[4.45906; 1546.40382]
Tharek_etal_2022	95	98	7	102	429.76190	[107.89447; 1711.81433]
Alis_etal_2022	105275	113288	16161	180278	133.41831	[129.75067; 137.18963]
Wang_etal_2021	207	219	9	231	425.50000	[175.65087; 1030.73927]
Ye_etal_2019	7533	34035	564	425688	214.25247	[196.50676; 233.60073]
Kuo_etal_2019	1131	1458	0	2939	20311.72061	[1266.71633; 325697.22612]
Arbabshirani_etal_2018	8739	13172	3745	33411	15.61605	[14.86021; 16.41034]
Majumdar_etal_2018	18	19	4	49	202.50000	[21.16372; 1937.57311]
Chang_etal_2018	7990	8038	239	2121	1310.77232	[957.49932; 1794.38672]
Grewal_etal_2018	59	131	8	199	19.56424	[8.90926; 42.96195]
Chilamkurthy_etal_2018	1985	2018	37	5728	9251.95332	[5770.58810; 14833.60772]
Random effects model		173426		662116	271.99909	[95.87258; 771.68582]
Heterogeneity: $I^2 = 100\%$, $\tau^2 = 3.5576$, $p = 0$						
g = 1						
Altuve_M_Pérez_2022a	96	100	4	100	576.00000	[139.99763; 2369.86869]
Rao_etal_2022a	555	559	17	605	4799.11765	[1604.93612; 14350.43422]
Zhou_etal_2022a	4358	6785	730	16256	38.19040	[34.92794; 41.75759]
Salehinejad_etal_2021a	2122	2288	306	15702	643.16702	[529.58739; 781.10586]
Kumaravel_etal_2021a	196	202	1	781	25480.00000	[3049.89288; 212869.90250]
Lee_etal_2019a	3166	3306	577	2128	60.78814	[50.04920; 73.83131]
Random effects model		13240		35572	606.02407	[87.29887; 4206.98657]
Heterogeneity: $I^2 = 99\%$, $\tau^2 = 5.5537$, $p < 0.01$						
g = 2						
F_Meng_etal_2022b	12	46	1	294	103.41176	[13.04035; 820.06940]
Trevisi_etal_2022b	201	276	58	533	21.94828	[15.00463; 32.10520]
XU_etal_2021b	51	67	4	203	158.57812	[50.81698; 494.85473]
Random effects model		389		1030	61.14423	[15.41809; 242.48254]
Heterogeneity: $I^2 = 83\%$, $\tau^2 = 1.1015$, $p < 0.01$						
g = 3						
F_Meng_etal_2022c	10	77	3	264	12.98507	[3.47629; 48.50342]
XU_etal_2021c	50	84	5	186	53.23529	[19.78870; 143.21284]
Kumaravel_etal_2021c	91	94	0	390	20417.57143	[1045.48164; 398741.79107]
Random effects model		255		840	199.15127	[2.93266; 13523.95657]
Heterogeneity: $I^2 = 90\%$, $\tau^2 = 12.9514$, $p < 0.01$						
**Random effects model**		**187310**		**699558**	**261.84412**	**[114.79759; 597.24549]**
Heterogeneity: $I^2 = 100\%$, $\tau^2 = 4.1617$, $p = 0$						
Test for subgroup differences: $\chi^2_3 = 4.43$, df = 3 ($p = 0.22$)						

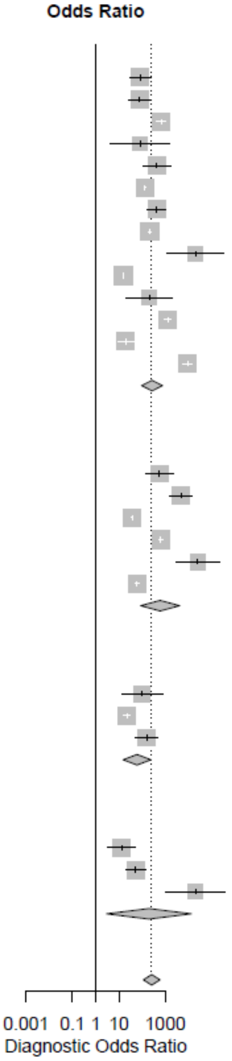

Figure S6. Univariate sub-group analysis of specificity with random model based on ICH types. G represents sub-group analysis of data, when g = 0 (EDH), g = 1 (SDH), g = 2 (IPH), g = 3 (IVH), g = 4 (SAH), and g = 5 (CPH).

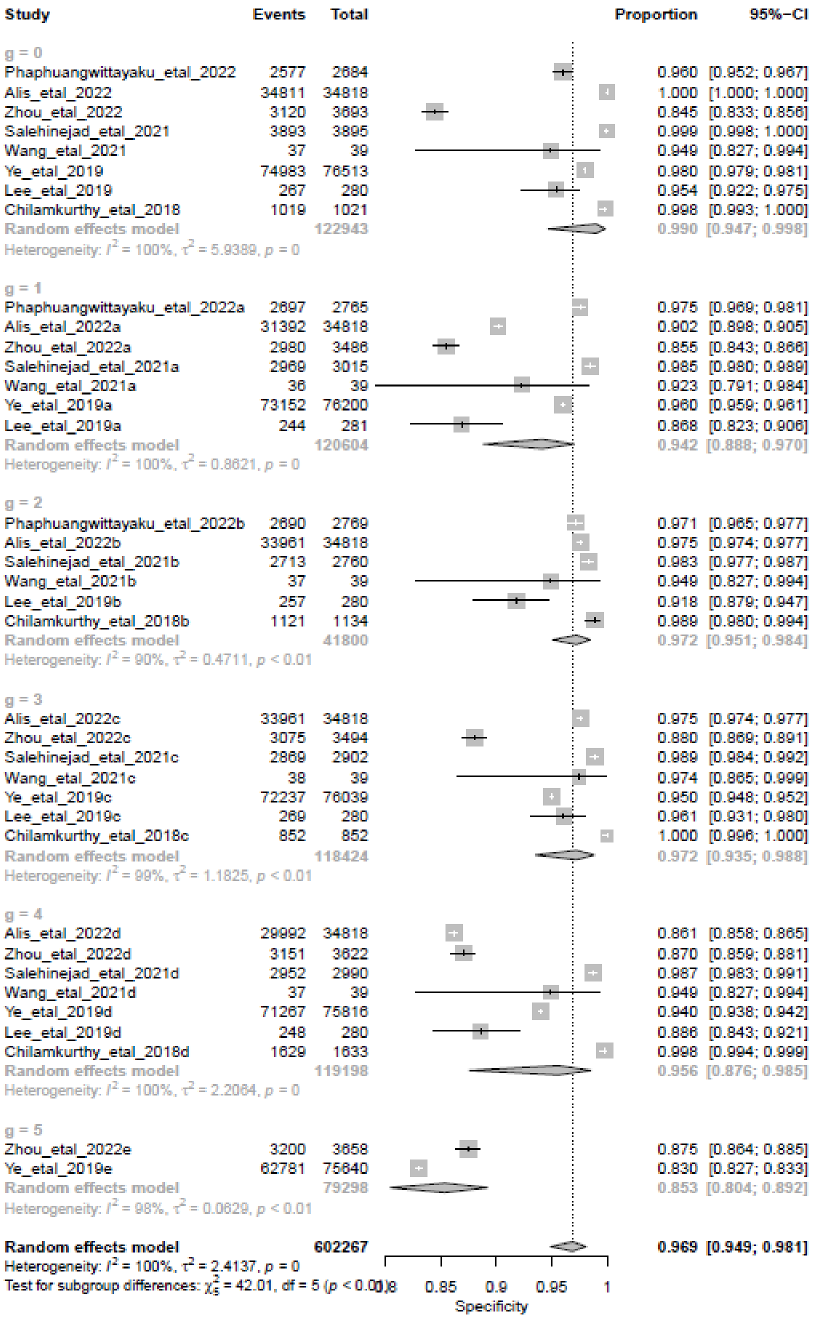

Figure S7. Univariate sub-group analysis of DOR with random model based on ICH types. G represents sub-group analysis of data, when g = 0 (EDH), g = 1 (SDH), g = 2 (IPH), g = 3 (IVH), g = 4 (SAH), and g = 5 (CPH).

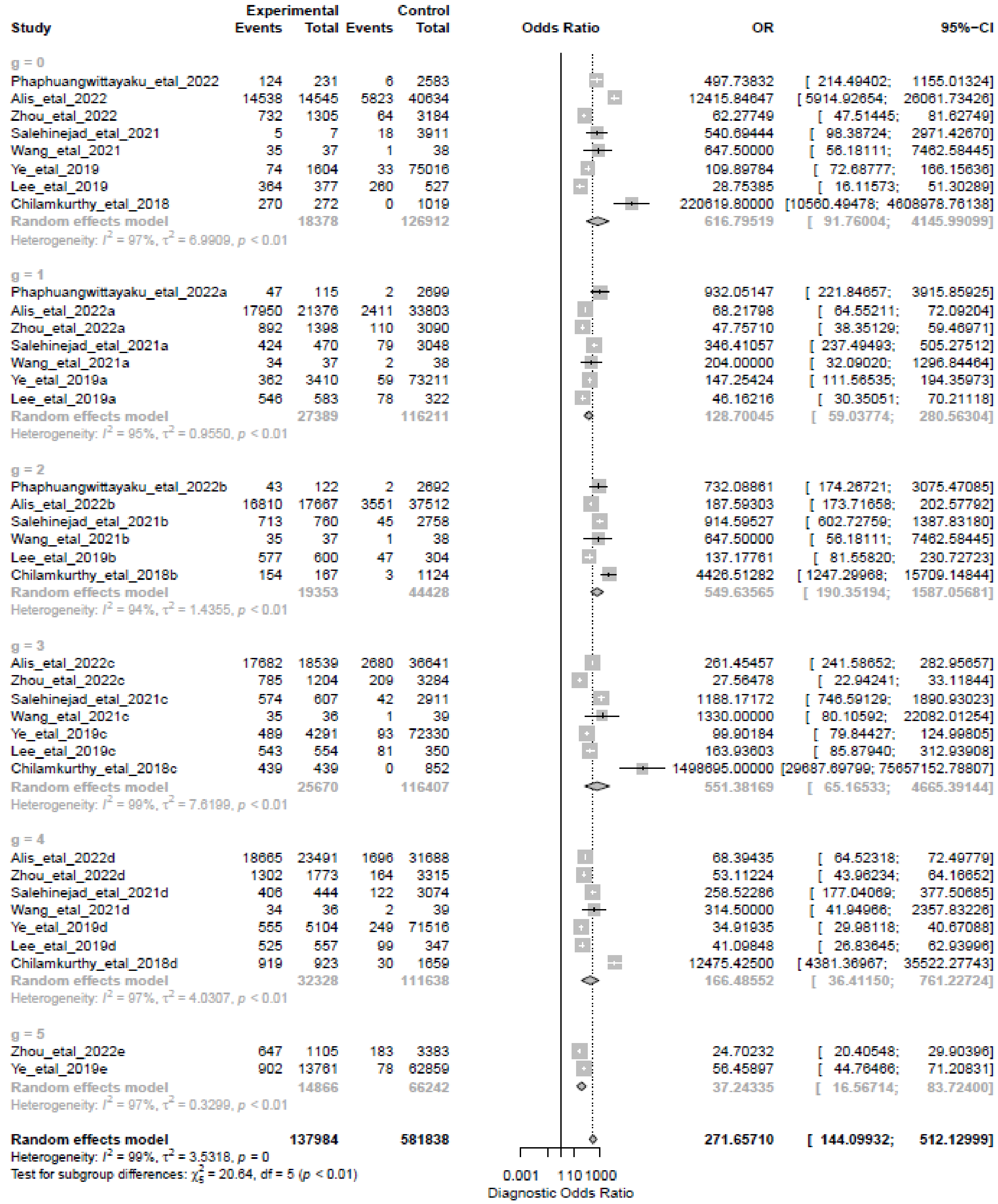

Figure S8. Univariate sub-group analysis of sensitivity with random model based on ICH types. G represents sub-group analysis of data, when g = 0 (EDH), g = 1 (SDH), g = 2 (IPH), g = 3 (IVH), g = 4 (SAH), and g = 5 (CPH).

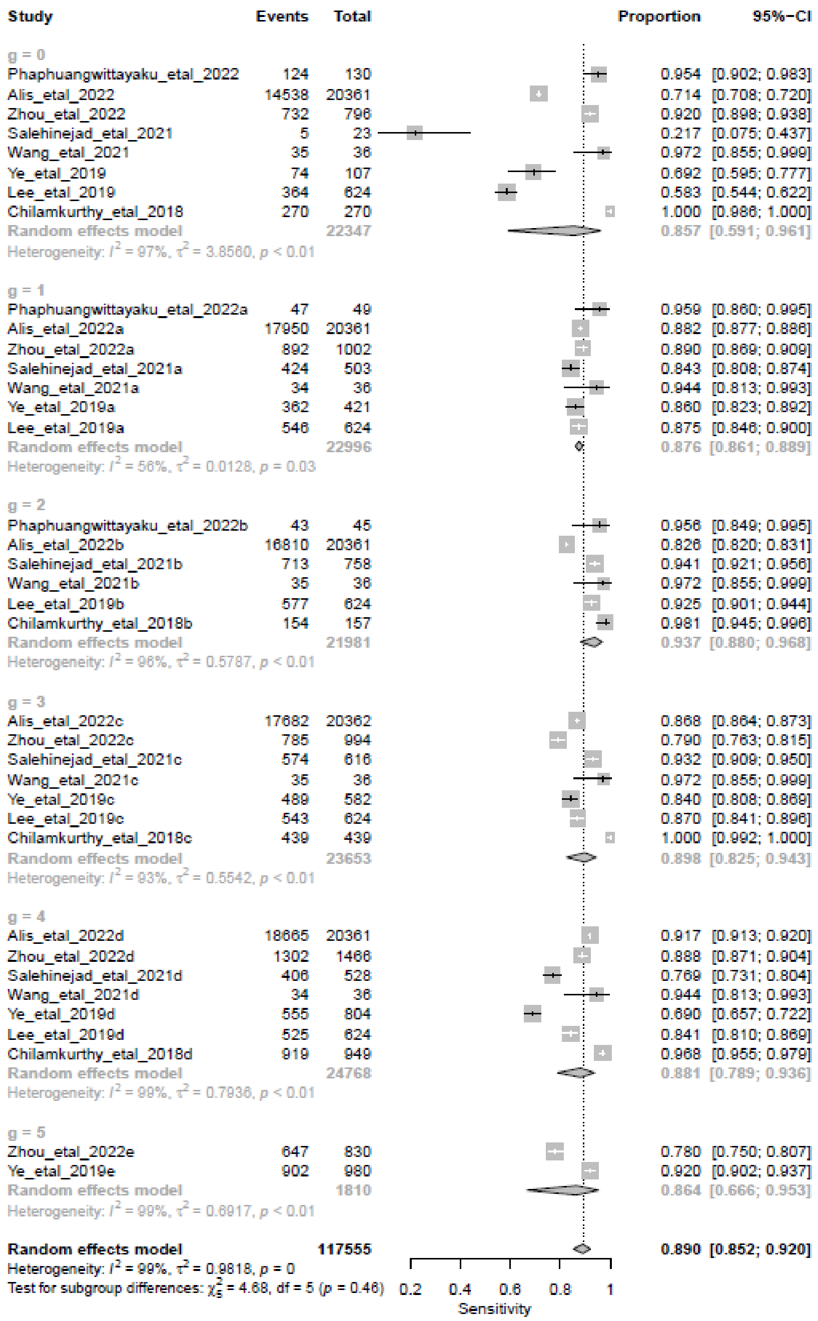

Figure S9. Univariate sub-group analysis of sensitivity with random model based on single or multiple center. G represents sub-group analysis of data, when g = 0 (Single), and g = 1 (Multiple).

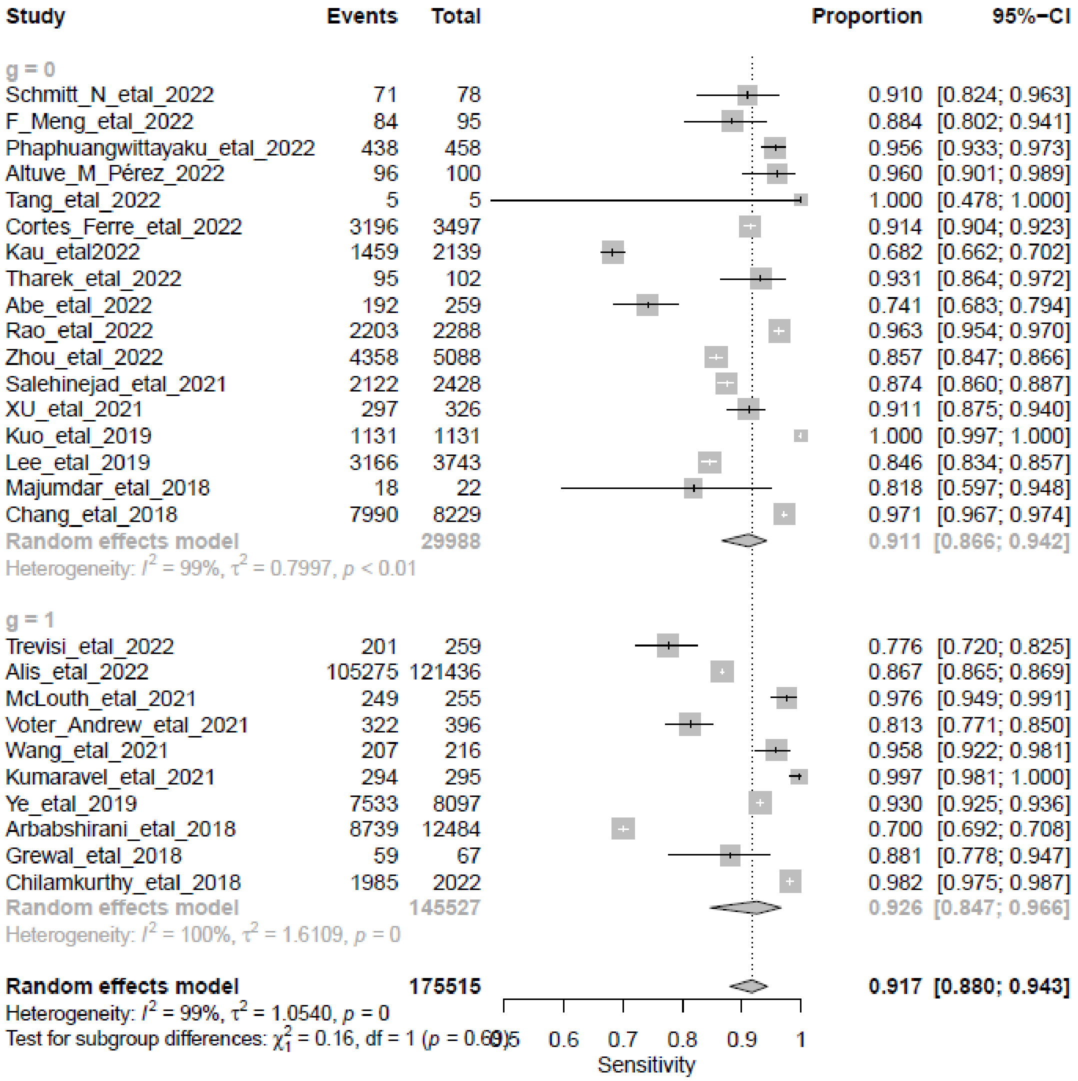

Figure S10. Univariate sub-group analysis of specificity with random model based on single or multiple center. G represents sub-group analysis of data, when g = 0 (Single), and g = 1 (Multiple).

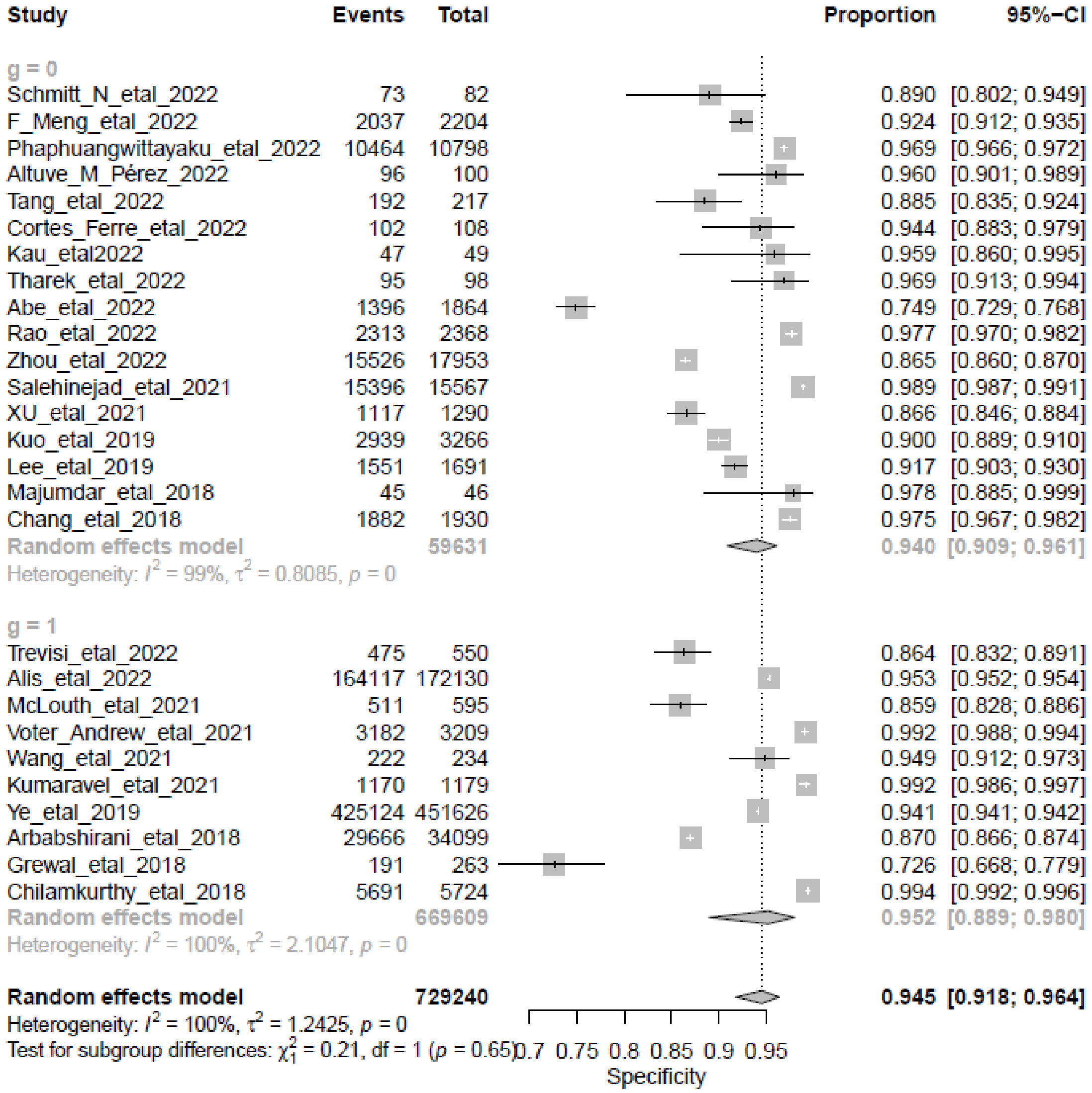

<u>Figure S11.</u> Univariate sub-group analysis of DOR with random model based on single or multiple center. G represents sub-group analysis of data, when g = 0 (Single), and g = 1 (Multiple).

Study	Experimental Events	Experimental Total	Control Events	Control Total	OR	95%-CI
g = 0						
Schmitt_N_etal_2022	71	80	7	80	82.26984	[29.06837; 232.84164]
F_Meng_etal_2022	84	251	11	2048	93.14535	[48.72575; 178.05895]
Phaphuangwittayaku_etal_2022	438	772	20	10484	686.11257	[432.60918; 1088.16569]
Altuve_M_Pérez_2022	96	100	4	100	576.00000	[139.99763; 2369.86869]
Tang_etal_2022	5	30	0	192	83.03922	[4.45906; 1546.40382]
Cortes_Ferre_etal_2022	3196	3202	301	403	180.50498	[78.56858; 414.69565]
Kau_etal2022	1459	1461	680	727	50.42132	[12.21185; 208.18383]
Tharek_etal_2022	95	98	7	102	429.76190	[107.89447; 1711.81433]
Abe_etal_2022	192	660	67	1463	8.54803	[6.35056; 11.50588]
Rao_etal_2022	2203	2258	85	2398	1089.95487	[772.58052; 1537.70588]
Zhou_etal_2022	4358	6785	730	16256	38.19040	[34.92794; 41.75759]
Salehinejad_etal_2021	2122	2293	306	15702	624.36097	[515.00236; 756.94142]
XU_etal_2021	297	470	29	1146	66.12498	[43.72735; 99.99490]
Kuo_etal_2019	1131	1458	0	2939	20311.72061	[1266.71633; 325697.22612]
Lee_etal_2019	3166	3306	577	2128	60.78814	[50.04920; 73.83131]
Majumdar_etal_2018	18	19	4	49	202.50000	[21.16372; 1937.57311]
Chang_etal_2018	7990	8038	239	2121	1310.77232	[957.49932; 1794.38672]
Random effects model		31281		58338	195.52692	[88.29934; 432.96790]
Heterogeneity: $I^2 = 99\%$, $\tau^2 = 2.4527$, $p = 0$						
g = 1						
Trevisi_etal_2022	201	276	58	533	21.94828	[15.00463; 32.10520]
Alis_etal_2022	105275	113288	16161	180278	133.41831	[129.75067; 137.18963]
McLouth_etal_2021	249	333	6	517	252.45833	[108.77401; 585.94153]
Voter_Andrew_etal_2021	322	349	74	3256	512.81481	[325.24708; 808.55157]
Wang_etal_2021	207	219	9	231	425.50000	[175.65087; 1030.73927]
Kumaravel_etal_2021	294	303	1	1171	38220.00000	[4823.12689; 302867.50314]
Ye_etal_2019	7533	34035	564	425688	214.25247	[196.50676; 233.60073]
Arbabshirani_etal_2018	8739	13172	3745	33411	15.61605	[14.86021; 16.41034]
Grewal_etal_2018	59	131	8	199	19.56424	[8.90926; 42.96195]
Chilamkurthy_etal_2018	1985	2018	37	5728	9251.95332	[5770.58810; 14833.60772]
Random effects model		164124		651012	265.59636	[57.73698; 1221.77208]
Heterogeneity: $I^2 = 100\%$, $\tau^2 = 5.8958$, $p = 0$						
**Random effects model**		**195405**		**709350**	**219.47017**	**[104.78742; 459.66547]**
Heterogeneity: $I^2 = 100\%$, $\tau^2 = 3.5521$, $p = 0$						
Test for subgroup differences: $\chi^2_1 = 0.12$, df = 1 ($p = 0.73$)						

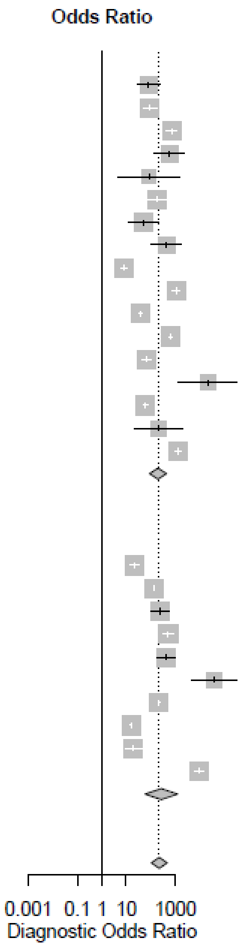

<u>Figure S12.</u> Univariate sub-group analysis of sensitivity with random model based on benchmark or real-time data. G represents sub-group analysis of data, when g = 0 (benchmark), and g = 1 (real-time data).

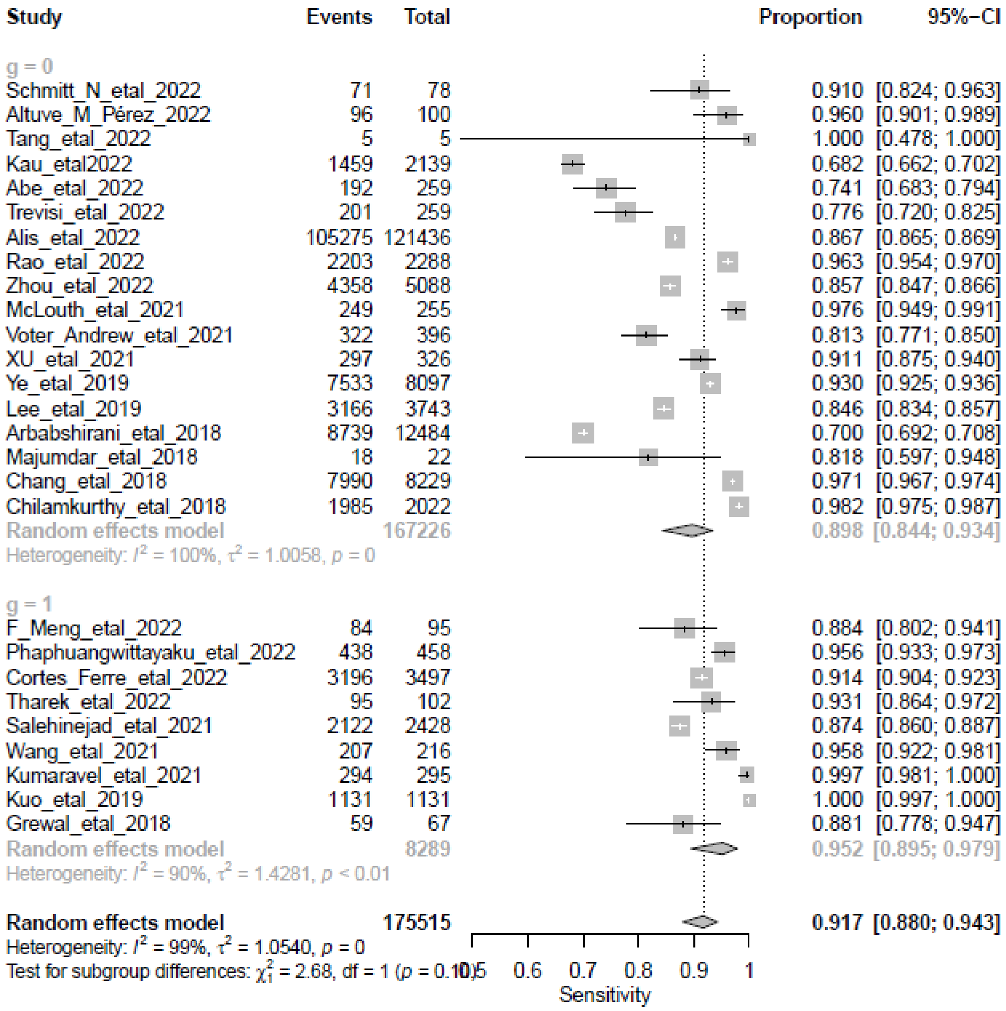

Figure S13. Univariate sub-group analysis of specificity with random model based on benchmark or real-time data. G represents sub-group analysis of data, when g = 0 (benchmark), and g = 1 (real-time data).

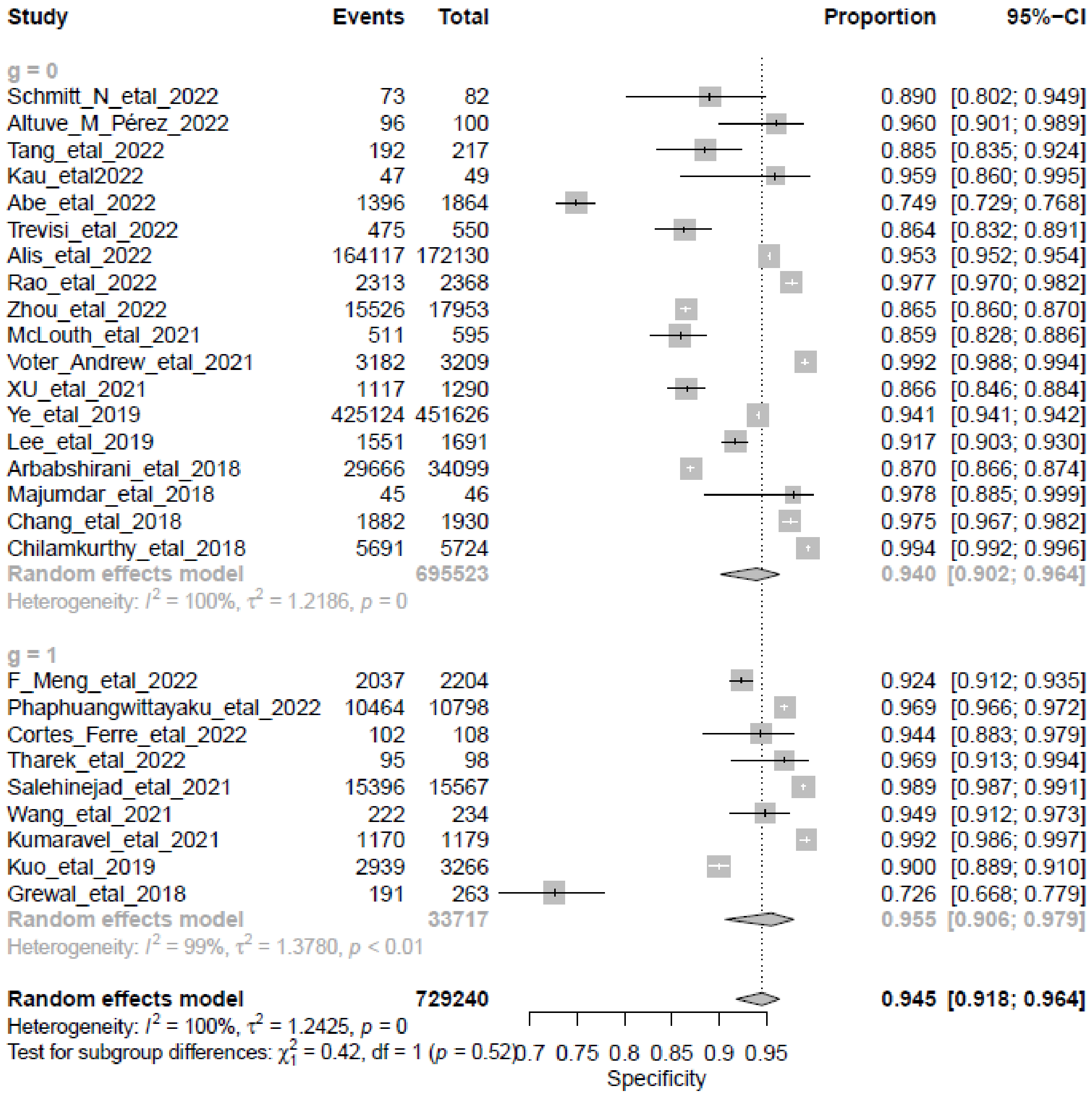

<u>Figure S14.</u> Univariate sub-group analysis of DOR with random model based on benchmark or real-time data. G represents sub-group analysis of data, when g = 0 (benchmark), and g = 1 (real-time data).

Study	Experimental Events	Experimental Total	Control Events	Control Total	OR	95%-CI
g = 0						
Schmitt_N_etal_2022	71	80	7	80	82.26984	[29.06837; 232.84164]
Altuve_M_Pérez_2022	96	100	4	100	576.00000	[139.99763; 2369.86869]
Tang_etal_2022	5	30	0	192	83.03922	[4.45906; 1546.40382]
Kau_etal2022	1459	1461	680	727	50.42132	[12.21185; 208.18383]
Abe_etal_2022	192	660	67	1463	8.54803	[6.35056; 11.50588]
Trevisi_etal_2022	201	276	58	533	21.94828	[15.00463; 32.10520]
Alis_etal_2022	105275	113288	16161	180278	133.41831	[129.75067; 137.18963]
Rao_etal_2022	2203	2258	85	2398	1089.95487	[772.58052; 1537.70588]
Zhou_etal_2022	4358	6785	730	16256	38.19040	[34.92794; 41.75759]
McLouth_etal_2021	249	333	6	517	252.45833	[108.77401; 585.94153]
Voter_Andrew_etal_2021	322	349	74	3256	512.81481	[325.24708; 808.55157]
XU_etal_2021	297	470	29	1146	66.12498	[43.72735; 99.99490]
Ye_etal_2019	7533	34035	564	425688	214.25247	[196.50676; 233.60073]
Lee_etal_2019	3166	3306	577	2128	60.78814	[50.04920; 73.83131]
Arbabshirani_etal_2018	8739	13172	3745	33411	15.61605	[14.86021; 16.41034]
Majumdar_etal_2018	18	19	4	49	202.50000	[21.16372; 1937.57311]
Chang_etal_2018	7990	8038	239	2121	1310.77232	[957.49932; 1794.38672]
Chilamkurthy_etal_2018	1985	2018	37	5728	9251.95332	[5770.58810; 14833.60772]
Random effects model		186678		676071	146.06044	[62.65084; 340.51662]
Heterogeneity: $I^2 = 100\%$, $\tau^2 = 3.1279$, $p = 0$						
g = 1						
F_Meng_etal_2022	84	251	11	2048	93.14535	[48.72575; 178.05895]
Phaphuangwittayaku_etal_2022	438	772	20	10484	686.11257	[432.60918; 1088.16569]
Cortes_Ferre_etal_2022	3196	3202	301	403	180.50498	[78.56858; 414.69565]
Tharek_etal_2022	95	98	7	102	429.76190	[107.89447; 1711.81433]
Salehinejad_etal_2021	2122	2293	306	15702	624.36097	[515.00236; 756.94142]
Wang_etal_2021	207	219	9	231	425.50000	[175.65087; 1030.73927]
Kumaravel_etal_2021	294	303	1	1171	38220.00000	[4823.12689; 302867.50314]
Kuo_etal_2019	1131	1458	0	2939	20311.72061	[1266.71633; 325697.22612]
Grewal_etal_2018	59	131	8	199	19.56424	[8.90926; 42.96195]
Random effects model		8727		33279	531.75250	[126.61610; 2233.21301]
Heterogeneity: $I^2 = 94\%$, $\tau^2 = 4.4102$, $p < 0.01$						
**Random effects model**		**195405**		**709350**	**219.47017**	**[104.78742; 459.66547]**
Heterogeneity: $I^2 = 100\%$, $\tau^2 = 3.5521$, $p = 0$						
Test for subgroup differences: $\chi^2_1 = 2.31$, df = 1 ($p = 0.13$)						

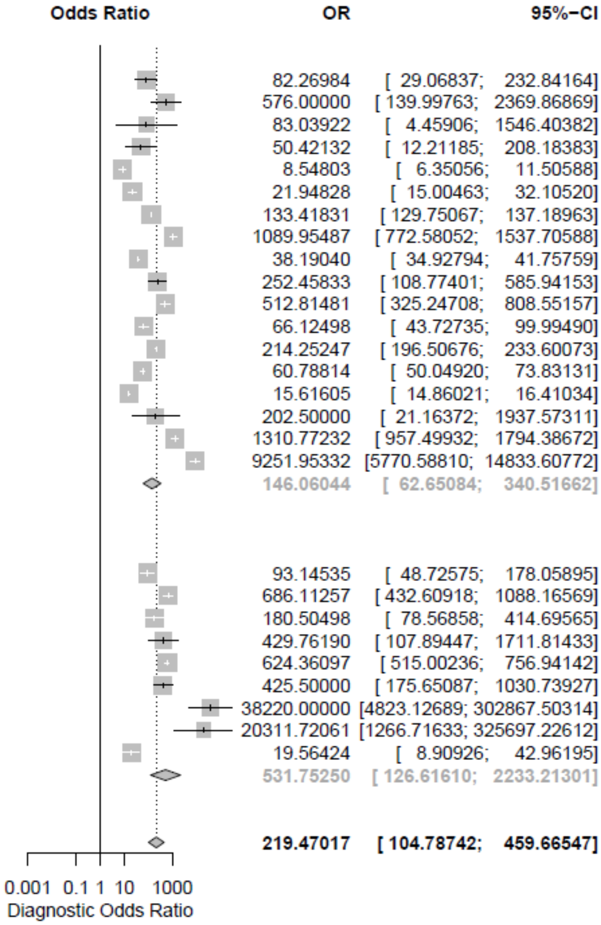

Supplement: Supplementary file 1 — Additional file 1: Figure S1. Univariate sub-group analysis of DOR with random model based on retrospective studies. Figure S2. Univariate sub-group analysis of DOR with random model based on prospective studies. Figure S3. Univariate sub-group analysis of specificity with random model based on Network Architecture. G represents sub-group analysis of data, when g = 0 (CNN), g = 1 (ResNet), g = 2 (RF), and g = 3 (SVM). Figure S4. Univariate sub-group analysis of sensitivity with random model based on Network Architecture. G represents sub-group analysis of data, when g = 0 (CNN), g = 1 (ResNet), g = 2 (RF), and g = 3 (SVM). Figure S5. Univariate sub-group analysis of DOR with random model based on Network Architecture. G represents sub-group analysis of data, when g = 0 (CNN), g = 1 (ResNet), g = 2 (RF), and g = 3 (SVM). Figure S6. Univariate sub-group analysis of specificity with random model based on ICH types. G represents sub-group analysis of data, when g = 0 (EDH), g = 1 (SDH), g = 2 (IPH), g = 3 (IVH), g = 4 (SAH), and g = 5 (CPH). Figure S7. Univariate sub-group analysis of DOR with random model based on ICH types. G represents sub-group analysis of data, when g = 0 (EDH), g = 1 (SDH), g = 2 (IPH), g = 3 (IVH), g = 4 (SAH), and g = 5 (CPH). Figure S8. Univariate sub-group analysis of sensitivity with random model based on ICH types. G represents sub-group analysis of data, when g = 0 (EDH), g = 1 (SDH), g = 2 (IPH), g = 3 (IVH), g = 4 (SAH), and g = 5 (CPH). Figure S9. Univariate sub-group analysis of sensitivity with random model based on single or multiple center. G represents sub-group analysis of data, when g = 0 (Single), and g = 1 (Multiple). Figure S10. Univariate sub-group analysis of specificity with random model based on single or multiple center. G represents sub-group analysis of data, when g = 0 (Single), and g = 1 (Multiple). Figure S11. Univariate sub-group analysis of DOR with random model based on single or multiple center. G represents sub-g [file 12938_2023_1172_MOESM1_ESM.pdf]
